# Gankyrin modulated non-small cell lung cancer progression via glycolysis metabolism in a YAP1-dependent manner

**DOI:** 10.1038/s41420-022-01104-3

**Published:** 2022-07-09

**Authors:** Tong Yu, Yanyan Liu, Junwen Xue, Xiang Sun, Di Zhu, Lu Ma, Yingying Guo, Tongzhu Jin, Huiying Cao, Yingzhun Chen, Tong Zhu, Xuelian Li, Haihai Liang, Zhimin Du, Hongli Shan

**Affiliations:** 1 Shanghai Frontiers Science Research Center for Druggability of Cardiovascular noncoding RNA, Institute for Frontier Medical Technology, Shanghai University of Engineering Science, Shanghai, 201620 P. R. China; 2 Department of Pharmacology (State-Province Key Laboratories of Biomedicine-Pharmaceutics of China, Key Laboratory of Cardiovascular Research, Ministry of Education), College of Pharmacy, Harbin Medical University, Harbin, Heilongjiang 150081 P. R. China; 3 Research Unit of Noninfectious Chronic Diseases in Frigid Zone (2019RU070), Chinese Academy of Medical Sciences, Harbin, Heilongjiang 150081 P. R. China; 4 Zhuhai People’s Hospital, Guangdong Provincial Key Laboratory of Tumor Interventional Diagnosis and Treatment, Zhuhai Hospital Affiliated with Jinan University, Jinan University, Zhuhai, Guangdong 519000 P. R. China; 5 Department of Pathology, the Second Affiliated Hospital, Harbin Medical University, Harbin, Heilongjiang 150081 P. R. China; 6 Department of General Surgery, the Fourth Affiliated Hospital, Harbin Medical University, Harbin, Heilongjiang 150081 P. R. China; 7 Institute of Clinical Pharmacy, the Second Affiliated Hospital, Harbin Medical University, Harbin, Heilongjiang 150081 P. R. China

**Keywords:** Oncogenes, Non-small-cell lung cancer

## Abstract

Non-small cell lung cancer (NSCLC) is highly malignant and heterogeneous form of lung cancer and involves various oncogene alterations. Glycolysis, an important step in tumor metabolism, is closely related to cancer progression. In this study, we investigated the biological function and mechanism of action of Gankyrin in glycolysis and its association with NSCLC. Analyzed of data from The Cancer Genome Atlas as well as NSCLC specimens and adjacent tissues demonstrated that Gankyrin expression was upregulated in NSCLC tissues compared to adjacent normal tissues. Gankyrin was found to significantly aggravate cancer-related phenotypes, including cell viability, migration, invasion, and epithelial mesenchymal transition (EMT), whereas Gankyrin silencing alleviated the malignant phenotype of NSCLC cells. Our results reveal that Gankyrin exerted its function by regulating YAP1 expression and increasing its nuclear translocation. Importantly, YAP1 actuates glycolysis, which involves glucose uptake, lactic acid production, and ATP generation and thus might contribute to the tumorigenic effect of Gankyrin. Furthermore, the Gankyrin-accelerated glycolysis in NSCLC cells was reversed by YAP1 deficiency. Gankyrin knockdown reduced A549 cell tumorigenesis and EMT and decreased YAP1 expression in a subcutaneous xenograft nude mouse model. In conclusion, both Gankyrin and YAP1 play important roles in tumor metabolism, and Gankyrin-targeted inhibition may be a potential anti-cancer therapeutic strategy for NSCLC.

## Introduction

Lung cancer remains the primary cause of cancer-related death and the second most common cause of human malignancy worldwide [1]. It is divided into non-small cell lung cancer (NSCLC) and small cell lung cancer. NSCLC accounts for approximately 85% of lung cancer [2]. Although targeted therapy and immune therapy can increase survival in many patients with advanced NSCLC, a considerable number of patients have poor prognosis and endure disease recurrence alongside drug resistance [3]. Therefore, it is necessary to explore the underlying molecular mechanisms of NSCLC tumorigenesis to identify promising targets for individualized therapies.

The energy metabolism process of multiple types of tumor cells is inconsistent with that of normal cells and involves aerobic glycolysis rather than more efficient mitochondrial oxidative phosphorylation [4]. This shift in energy generation supports the vigorous growth and metastasis of tumors. Glycolysis is a cascade reaction that is catalyzed by a series of enzymes, including hexokinase 2 (HK2), phosphoglycerate kinase 1 (PGK1), the M2 isoform of pyruvate kinase (PKM2), and lactate dehydrogenase A (LDHA) [5], which together generate ATP by consuming glucose and producing lactic acid [6]. Thus, interfering with the glycolytic process is gradually being targeted as a new anti-cancer strategy. Reportedly, EGFR inhibitors can reverse glycolysis and reactivate mitochondrial oxidative phosphorylation by inhibiting HK2 activity and reducing PKM2 phosphorylation [7]. The tumor-associated glycoprotein CD147, which regulates cell metabolism, is methylated by the lysine methyltransferase KMT5A and promotes glycolysis and lactic acid production in NSCLC cells, thereby affecting cancer progression and overall survival [8].

The Gankyrin gene (*PSMD10*) is localized on human chromosome Xq22.3. Gankyrin has been primarily identified as an oncoprotein in human hepatocellular carcinoma [9]. Increasing evidence has revealed an important role of Gankyrin in the development of several other human cancers. Gankyrin activates the PI3K/AKT/HIF-1α/cyclin D1 pathway, thereby accelerating ovarian cancer cell proliferation [10]. Luo et al. have found that Gankyrin induces autophagy to promote tumor progression through ATG7 [11]. Furthermore, Gankyrin was found to stimulate lung cancer metastasis through a closed circle with in the IL-6/p-STAT3 and TGF-β/p-SMAD3 signaling pathways [12]. However, the potential regulatory mechanism by which Gankyrin in mediates cell metabolism, including glycolysis, in NSCLC is not well understood.

This study illustrated the upregulation of Gankyrin expression in NSCLC tissues compared to adjacent non-tumor tissues. Specifically, we found that Gankyrin accelerated the proliferation and invasion of NSCLC by promoting the nuclear localization of YAP1 and activating glycolysis-related kinases. Silencing Gankyrin was found to blunt the EMT process in NSCLC cells and suppress tumor growth in vivo. Gankyrin may therefore be a potential target for metabolic reprogramming in NSCLC treatment.

## Results

### Gankyrin expression is frequently increased in NSCLC

**Fig. 1:**
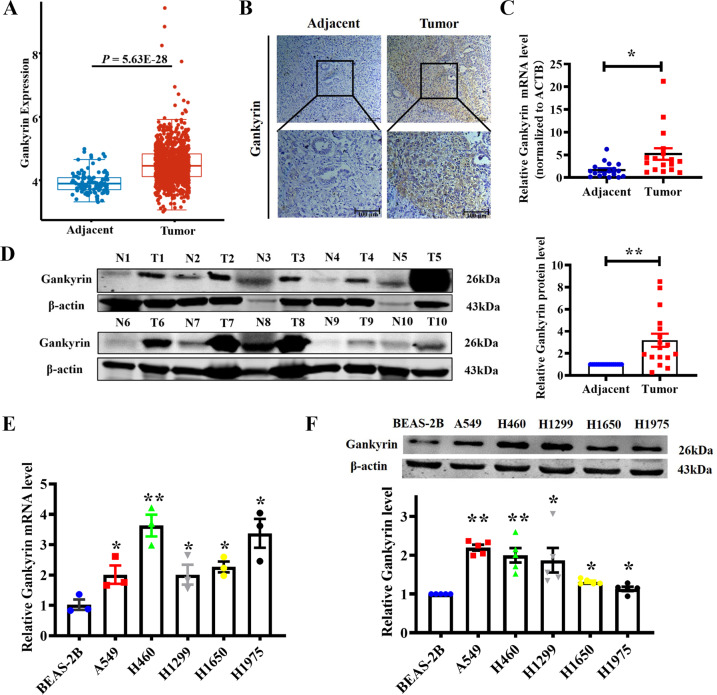
Expression of Gankyrin is upregulated in NSCLC. **A** The expression of Gankyrin in NSCLC tissues and adjacent normal tissues in TCGA dataset (*P* = 5.63E-28, Student’s *t*-test). **B** Representative images of Gankyrin immunohistochemistry staining in NSCLC and adjacent normal tissues (Scale bars: 100 μm). **C** qRT-PCR was performed to assess the mRNA level of Gankyrin in tumor tissues and adjacent tissues (*n* = 17, *P < 0.05). **D** Gankyrin protein levels in tumor tissues and adjacent tissues evaluated through western blotting. T: tumors; N: adjacent normal tissues (*n* = 17, **P < 0.01). **E** The mRNA level of Gankyrin in BEAS-2B, A549, H460, H1299, H1650, and H1975 cells assessed using qRT-PCR (*n* = 3, *P < 0.05, **P < 0.01 *vs*. BEAS-2B). **F** Western blotting analysis of Gankyrin expression in BEAS-2B, A549, H460, H1299, H1650 and H1975 cells (*n* = 5, *P < 0.05, **P < 0.01 *vs*. BEAS-2B).

First, we investigated the differential expression of Gankyrin in NSCLC using TCGA database. As shown in Fig. 1A, Gankyrin expression was higher in NSCLC than in adjacent tissues. Next, we collected discarded samples from NSCLC patients after the pathological examination following surgery to examine the protein and mRNA levels of Gankyrin. Immunohistochemical staining revealed that tumor tissues were positive for Gankyrin expression, with lower levels observed in adjacent tissues than in tumor tissues (Fig. 1B). The mRNA and protein levels of Gankyrin were also higher in tumor tissues than adjacent tissues (Fig. 1C, D). Subsequently, we tested the Gankyrin expression in different NSCLC cell lines and BEAS-2B cells. Both mRNA and protein Gankyrin levels were substantially higher in the A549, H460, H1299, H1650, and H1975 cell lines than those in BEAS-2B cells (Fig. 1E, F).

### Gankyrin aggravates the malignant phenotypes of NSCLC cells and induces EMT

**Fig. 2:**
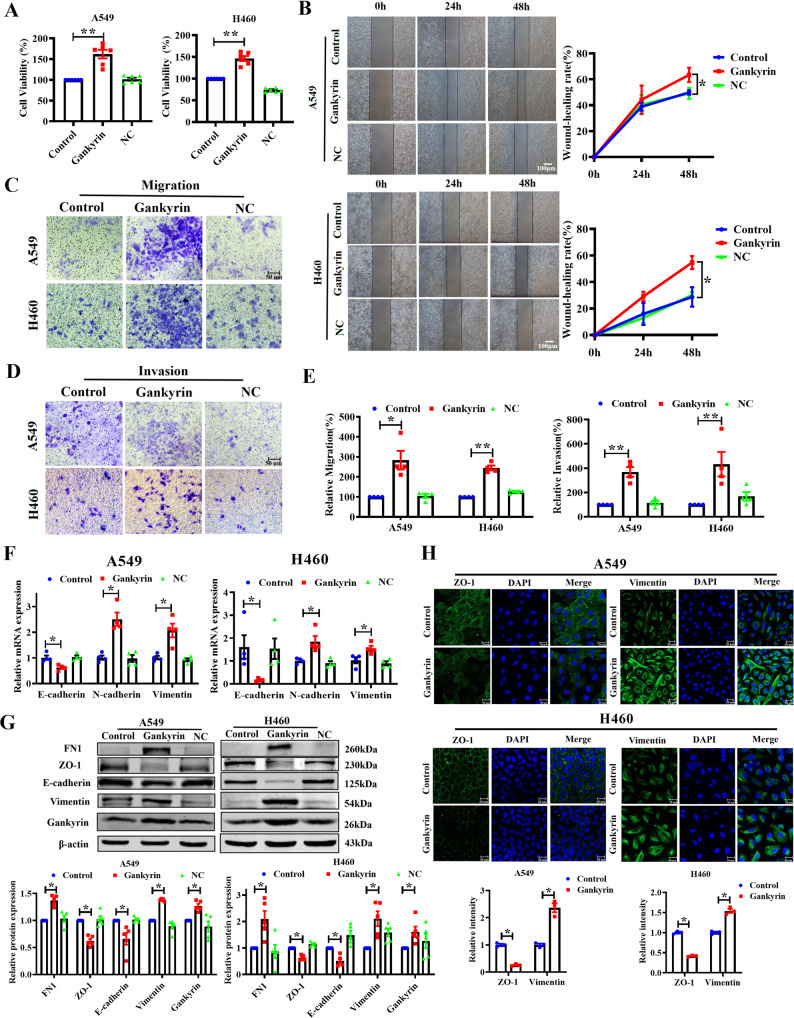
Overexpression of Gankyrin promotes NSCLC cell proliferation, migration, invasion, and EMT. **A** MTT assay was used to detect the viability of the NSCLC cells A549 and H460 (*n* = 6, **P < 0.01). **B** Wound-healing assays demonstrated the migration ability of A549 and H460 cells (*n* = 5, *P < 0.05. Scale bars: 100 μm). **C**, **D** Migration and invasion experiments performed for A549 and H460 cells through transwell assay (Scale bars: 50 μm). **E** Statistical analysis of migration and invasion assay (*n* = 4, *P < 0.05, **P < 0.01). **F** qRT-PCR assay showed that the mRNA level of EMT-relevant markers in A549 and H460 cells (*n* = 4, *P < 0.05). **G** Western blotting assay showed the protein expression of EMT-relevant markers in A549 and H460 cells (*n* = 5, *P < 0.05). **H** Immunofluorescence assay was used to detect the Vimentin and ZO**-**1 in A549 and H460 cells with the overexpression of Gankyrin (Scale bars: 20 μm). Vimentin and ZO-1 intensity assays were shown at the bottom of the image (*n* = 3, *P < 0.05).

To determine the functions of Gankyrin in NSCLC, A549 and H460 cells were transfected with a Gankyrin-overexpression plasmid. The MTT assay showed that Gankyrin increased the proliferation of A549 and H460 cells (Fig. 2A), while the wound-healing assay showed that Gankyrin promoted the migration of NSCLC cells, with similar cell migration observed in the negative control (NC) and control groups (Fig. 2B). Transwell migration and Matrigel invasion assays indicated that Gankyrin overexpression significantly promoted the migratory and invasive capacities of the A549 and H460 cells (Fig. 2C–E).

EMT refers to the biological process by which epithelial cells transform into cells with a mesenchymal phenotype through specific procedures [13] and plays an important role in tumor invasion and metastasis. Herein, the effect that Gankyrin has on the EMT process was therefore examined. The results of qRT-PCR and western blotting showed that Gankyrin overexpression decreased the expression of the epithelial markers E-cadherin and ZO-1, and promoted the expression of the mesenchymal markers FN1, Vimentin, and N-cadherin in the A549 and H460 cells (Fig. 2F, G). Immunofluorescence analysis further confirmed that Gankyrin promoted EMT (Fig. 2H).

### Silencing Gankyrin suppresses the proliferation, migration, invasion, and EMT in NSCLC cells

**Fig. 3:**
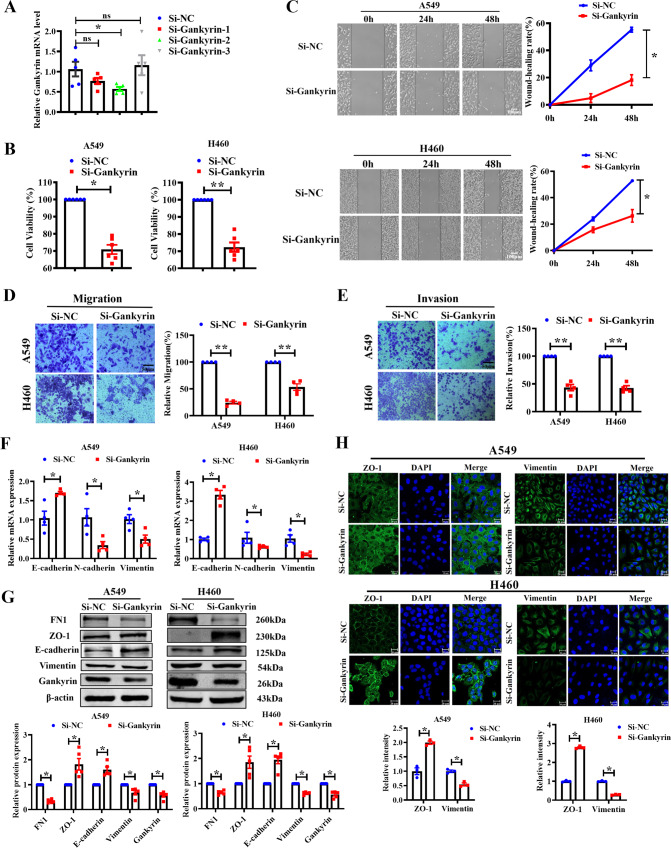
Gankyrin knockdown inhibits malignant phenotypes of NSCLC cells in vitro. **A** Real-time PCR analysis verified the efficiency of Gankyrin knockdown (*n* = 5, *P < 0.05, ns means no significant). **B** Viability of A549 and H460 cells as determined using MTT assay (*n* = 6, *P < 0.05, **P < 0.01). **C** Wound-healing assays showing cell migration in A549 and H460 cells (*n* = 5, *P < 0.05, Scale bars: 100 μm). **D** Migration assays in A549 and H460 cells, respectively (*n* = 4, **P < 0.01, Scale bars: 50 μm). **E** The invasion of A549 and H460 cells was determined via invasion assay using Matrigel transwell chambers (*n* = 4, **P < 0.01, Scale bars: 50 μm). **F** mRNA levels of EMT-relevant markers in A549 and H460 cells (*n* = 4, *P < 0.05). **G** Levels of the EMT-relevant markers E-cadherin, FN1, ZO-1, and Vimentin in A549 and H460 cells (*n* = 5, *P < 0.05). **H** Immunofluorescence assay was used to detect the Vimentin and ZO-1 levels in A549 and H460 cells (Scale bars: 20 μm). Vimentin and ZO-1 intensity assays were shown at the bottom of the image (*n* = 3, *P < 0.05).

To examine whether silencing Gankyrin could alleviate the malignancy of NSCLC cells, Gankyrin was knocked down in A549 and H460 cells using siRNA. Three siRNAs were designed and qRT-PCR was used to verify a knockdown efficiency of approximately 50% for si-Gankyrin-2, which was then utilized in subsequent experiments (Fig. 3A). Silencing Gankyrin significantly reduced the viability of NSCLC cells (Fig. 3B), and wound-healing assay showed that si-Gankyrin inhibited the migration of NSCLC cells (Fig. 3C). Transwell migration and Matrigel invasion assays indicated that Gankyrin silencing significantly alleviated the migratory and invasive capacities of the A549 and H460 cells (Fig. 3D–E). Additionally, Gankyrin silencing markedly increased the E-cadherin and ZO-1 expression, and decreased the expression of FN1, Vimentin, and N-cadherin (Fig. 3F–H).

### Gankyrin promotes the expression and nuclear translocation of YAP1

**Fig. 4:**
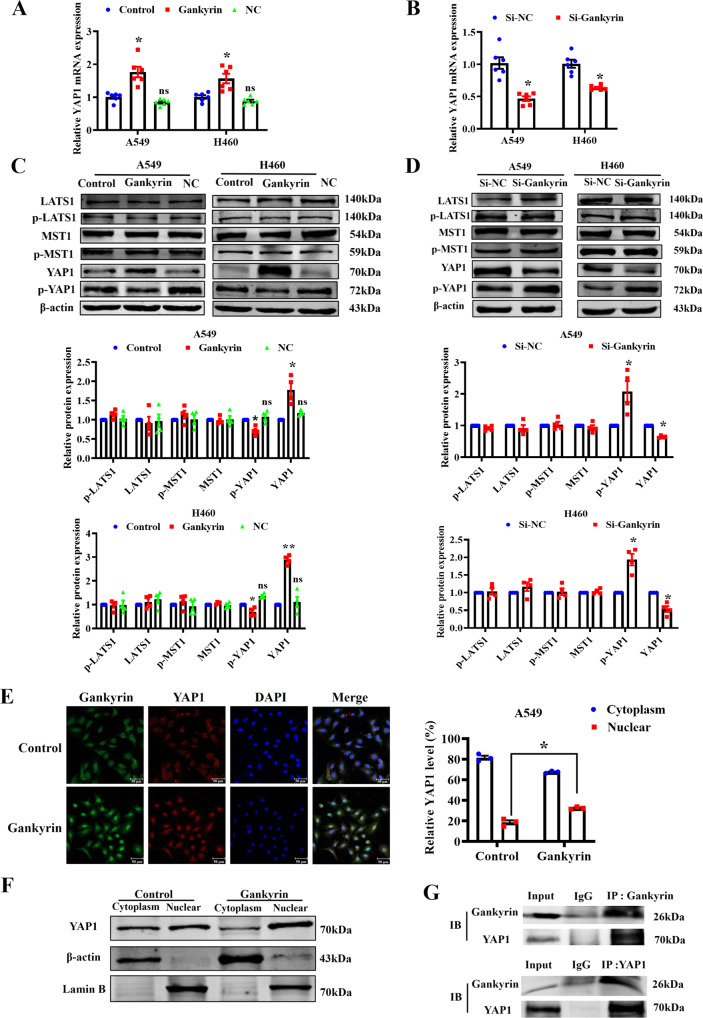
Gankyrin promotes YAP1 nuclear translocation. **A**, **B** qRT-PCR assay results show the mRNA levels of *YAP1* in A549 and H460 cells (*n* = 6, *P < 0.05 *vs*. Control or Si-NC). **C**, **D** Western blotting assay results show the protein levels of LATS1, p-LATS1, MST1, p-MST1, YAP1, p-YAP1 in A549 and H460 cells (*n* = 4, *P < 0.05, **P < 0.01 *vs*. Control or Si-NC). **E** Representative immunofluorescence images of Gankyrin and YAP1 in A549 (*n* = 3, *P < 0.05, Scale bars: 50 μm). **F** Western blotting shows YAP1 expression in the nuclear fraction and cytoplasm of A549 cells. **G** Immunoprecipitation assay indicates interaction between Gankyrin and YAP1 in A549.

Gankyrin acts as an oncogene in osteosarcoma carcinogenesis by regulating the miR-200a-p53-YAP1 loop [14]. Our previous study showed that YAP1 promotes NSCLC tumorigenesis and metastasis by regulating Slug transcription in a YAP1/TEAD-dependent manner [15]. We hypothesized that Gankyrin facilitates NSCLC tumorigenesis by regulating YAP1 expression. qRT-PCR results showed that *YAP1* mRNA levels was increased in cells transfected with Gankyrin-overexpression plasmid (Fig. 4A). In contrast, silencing Gankyrin inhibited the mRNA level of *YAP1* (Fig. 4B). The protein expression level of YAP1 was consistent with that in the qRT-PCR data. Gankyrin overexpression inhibited the phosphorylation of YAP1 but had no effect on the phosphorylation levels of LATS1 and MST1 (Fig. 4C, Supplementary Fig. 1A, B). In contrast, silencing Gankyrin promoted YAP1 phosphorylation (Fig. 4D, Supplementary Fig. 1C, D). While assessing the relationship between Gankyrin and YAP1, we observed that Gankyrin overexpression significantly promoted YAP1 nuclear translocation compared to the control group (Fig. 4E). Consistent with this results, YAP1 subcellular localization assay showed higher YAP1 accumulation in the nucleus and lower YAP1 levels in the cytoplasm of Gankyrin-overexpressing cells (Fig. 4F). Furthermore, co-immunoprecipitation assays indicated that Gankyrin interacted with YAP1 (Fig. 4G). These results indicate that Gankyrin binds to YAP1 and promotes its nuclear translocation.

### YAP1 is necessary for Gankyrin-mediated NSCLC tumorigenesis and EMT

**Fig. 5:**
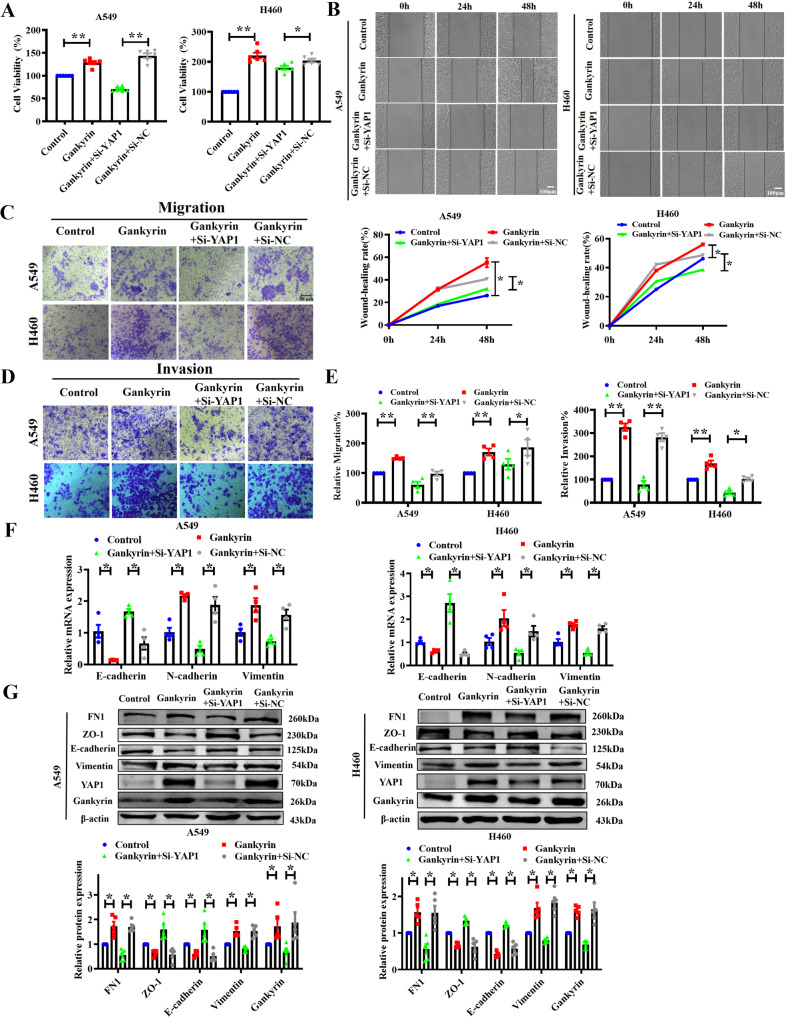
Gankyrin promotes EMT progression through YAP1 regulation. **A** Viability of A549 and H460 cells determined using MTT assay (*n* = 6, *P < 0.05, **P < 0.01). **B** Wound-healing assays show the motility of A549 and H460 cells (*n* = 5, *P < 0.05, Scale bars: 100 μm). **C**, **D** Representative images of transwell migration and Matrigel invasion assays in A549 and H460 cells (Scale bars: 50 μm). **E** Statistical analysis of migration and invasion in A549 and H460 cells (*n* = 4, *P < 0.05, **P < 0.01). **F** mRNA levels of the EMT-relevant markers E-cadherin, N-cadherin, and Vimentin in A549 and H460 cells (*n* = 4, *P < 0.05). **G** Protein levels of the EMT-relevant markers E-cadherin, N-cadherin, and Vimentin in A549 and H460 cells (*n* = 5, *P < 0.05).

To validate the role of YAP1 in Gankyrin-mediated NSCLC tumorigenesis and EMT, A549 and H460 cells were transfected with YAP1 siRNA. We found that silencing YAP1 attenuated the Gankyrin-induced cell viability (Fig. 5A). Wound-healing assay showed that knockdown of YAP1 reversed Gankyrin-induced cell migration of A549 and H460 cells (Fig. 5B). The transwell migration and Matrigel invasion assays showed that YAP1 silencing alleviated the migratory and invasive capacities of NSCLC cells that were promoted by Gankyrin (Fig. 5C–E). Next, qRT-PCR and western blotting showed that knockdown of YAP1 markedly mitigated the decrease in E-cadherin and ZO-1 expression while inhibiting the increase of N-cadherin, FN1, and Vimentin induced expression by Gankyrin overexpression in A549 and H460 cells (Fig. 5F, G). These results indicate that YAP1 mediates the Gankyrin-promoting effect in NSCLC tumorigenesis.

### Gankyrin accelerates glycolysis in NSCLC cells

**Fig. 6:**
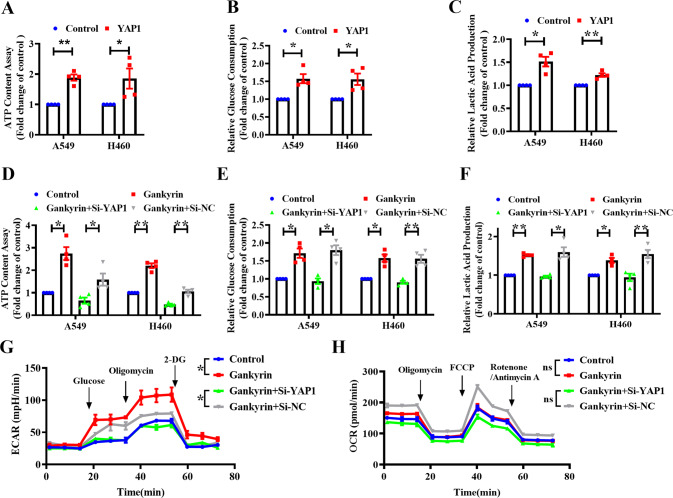
Gankyrin promotes glycolysis through YAP1. Relative ATP content (**A**, **D**), glucose consumption (**B**, **E**), and lactic acid production (**C**, **F**) were determined in A549 and H460 cells transfected with YAP1-overexpression plasmid or with Gankyrin-overexpression plasmid and YAP1 siRNA or si-NC (*n* = 4, *P < 0.05, **P < 0.01). **G** ECAR was measured using a Seahorse Bioscience Extracellular Flux Analyzer after transfection with Gankyrin and si-YAP1 in A549 cells. ECAR curves were obtained after treatment with glucose, oligomycin, and 2-DG. Black arrows represent the time of cell treatment (*n* = 3, *P < 0.05). **H** OCR was measured using Seahorse Bioscience Extracellular Flux Analyzer after transfecting A549 cells with Gankyrin and si-YAP1. OCR curves were obtained after treatment with oligomycin, FCCP, and rotenone/antimycin A. Black arrows indicate the time of treatment (*n* = 3, ns means no significant).

Gankyrin has been reported to drive glycolysis and glutaminolysis in hepatocellular carcinoma by upregulating c-Myc expression via the activation of β-catenin signaling [16]. We assumed that Gankyrin also affects glycolysis in NSCLC cells by interacting with YAP1. To verify this hypothesis, the cellular ATP content, glucose consumption, and lactic acid production were measured. Overexpression of YAP1 increased the ATP content, glucose consumption, and lactic acid production in A549 and H460 cells (Fig. 6A–C), while YAP1 knockdown inhibited these processes (Supplementary Fig. 2A–C). Consistently, forced Gankyrin expression significantly upregulated the ATP content, glucose consumption, and lactic acid production (Fig. 6D–F), which were downregulated by YAP1 siRNA co-transfection. To further assess the effects of Gankyrin and YAP1 on the regulation of glycolysis and mitochondrial oxidative phosphorylation, we measured the ECAR and OCR in A549 cells. The ECAR measurement assay showed that Gankyrin overexpression increased the glycolytic capacity and reserve in A549 cells, whereas, silencing YAP1 partly suppressed the glycolysis rate (Fig. 6G). Interestingly, no significant differences were observed in the parameters describing OCR (Fig. 6H).

**Fig. 7:**
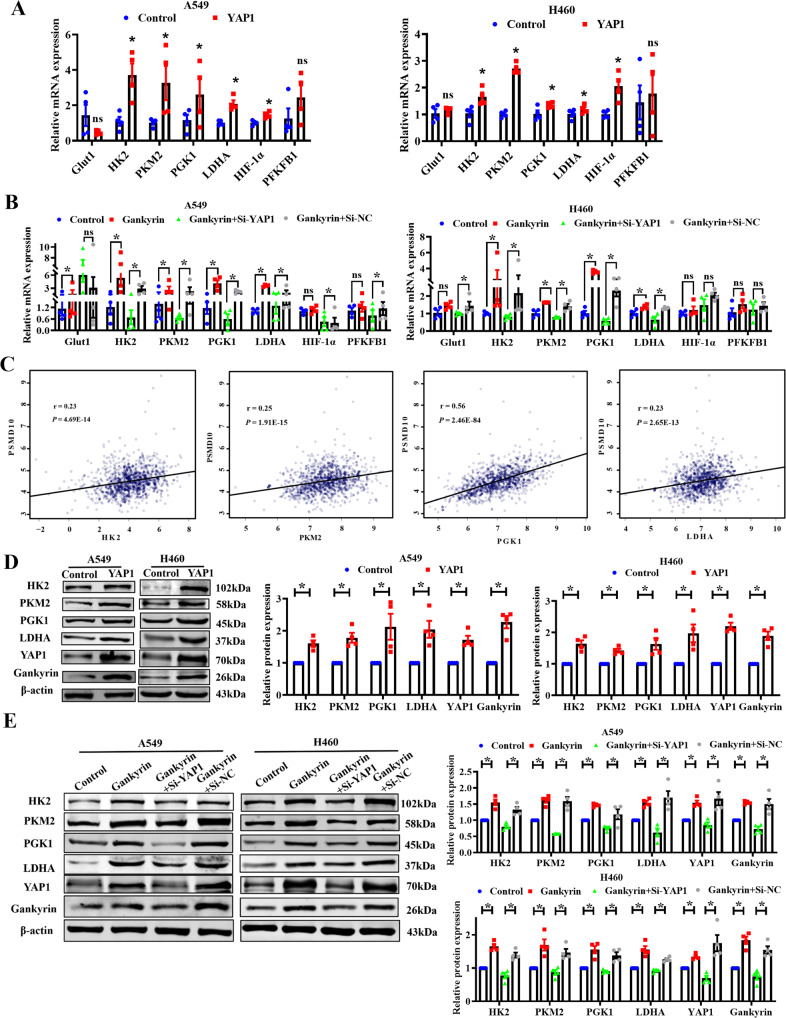
Gankyrin regulates the key enzymes of glycolysis via YAP1 in NSCLC cells. **A**, **B** qRT-PCR assay results show the mRNA levels of glycolytic genes including *Glut1*, *HK2*, *PKM2*, *PGK1*, *LDHA*, *HIF-1α*, and *PFKFB1* in A549 and H460 cells (*n* = 4, *P < 0.05, ns means no significant). **C** Correlation between expression of *PSMD10* and the glycolytic genes HK2, LDHA, PGK1, and PKM2 in TCGA dataset. **D**, **E** Western blotting results show the protein level of key glycolysis enzymes, including HK2, PKM2, PGK1, and LDHA in A549 and H460 cells (*n* = 4, *P < 0.05).

To further explore how Gankyrin regulates cellular glycolysis, the mRNA levels of glucose transporter and several metabolic enzymes were measured. We found that YAP1 partially increased the expression of glycolysis-related enzymes (Fig. 7A), while YAP1 knockdown decreased the mRNA levels of *HK2*, *PKM2*, *PGK1*, and *LDHA* but did not affect *GLUT1*, *HIF-1α*, or *PFKFB1* (Supplementary Fig. 2D–E). Furthermore, the depletion of YAP1 by siRNA resulted in the suppression of glycolysis-associated enzymes that were induced by Gankyrin overexpression, suggesting the involvement of YAP1 in the above process (Fig. 7B). Correlation analysis between genes showing obvious changes indicated an association between Gankyrin (*PSMD10*) and glycolysis-related enzymes. Gankyrin expression was positively correlated with the expression levels of glycolytic genes including *HK2*, *LDHA*, *PGK1*, and *PKM2* (Fig. 7C, *P* < 0.05, Pearson’s correlation test). Furthermore, the results of the western blotting assay revealed that the overexpression of Gankyrin and YAP1 upregulated the protein expression of HK2, PKM2, PGK1, and LDHA, which was then reversed by silencing YAP1 (Fig. 7D–E). In addition, YAP1 knockdown decreased the protein expression of HK2, PKM2, PGK1, and LDHA in A549 and H460 cells (Supplementary Fig. 2F, G). These data support the hypothesis that Gankyrin regulates glycolysis via YAP1 in NSCLC cells.

### Gankyrin knockdown inhibits tumorigenesis of A549 cells in vivo

**Fig. 8:**
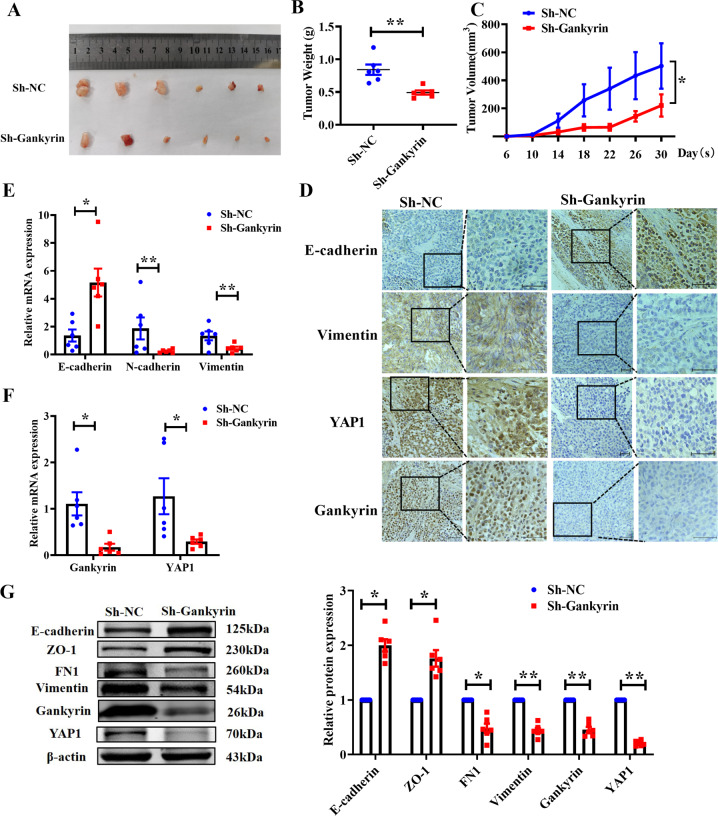
Gankyrin knockdown inhibits EMT and tumor progression in vivo. **A** Macroscopic observation of the size of tumor nodules in nude mice bearing xenograft tumors with A549-sh-Gankyrin or sh-NC. **B** Statistical analysis of tumor weight in different groups (*n* = 6, **P < 0.01). **C** Statistical analysis of tumor growth curves in different group (*n* = 6, *P < 0.05). **D** Immunohistochemistry assay was applied to determine the expression of E-cadherin, Vimentin, YAP1, and Gankyrin in a cell-derived xenograft tumor model (Scale bars: 100 μm). **E**, **F** Expression of Gankyrin, and YAP1, and EMT-relevant markers, including E-cadherin, N-cadherin, and Vimentin in different groups, as determined by using qRT-PCR (*n* = 6, *P < 0.05, **P < 0.01). **G** Western blotting assay results show the protein levels of the EMT-relevant markers E-cadherin, ZO-1, FN1, N-cadherin, and Vimentin (*n* = 6, *P < 0.05, **P < 0.01).

To better understand the role of Gankyrin in tumorigenesis, stable Gankyrin-knockdown A549 cells (LV-h-Gankyrin-shRNA) and NC A549 cells (LV-h-NC-shRNA) were generated. Nude mice were subcutaneously injected with A549-sh-Gankyrin cells to construct xenograft models, and tumor growth was observed for four weeks. As shown in Fig. 8A, Gankyrin knockdown significantly restrained the growth of tumors as compared to mice injected with the A549-sh-NC cells. Moreover, A549-sh-Gankyrin cells decreased the tumor weight and volume in the xenograft models (Fig. 8B, C). Immunohistochemical staining of EMT markers showed that tumors from the sh-Gankyrin group exhibited relatively high E-cadherin expression and low Vimentin expression compared to the sh-NC group. In addition, Gankyrin knockdown resulted in a conspicuous decrease in YAP1 staining (Fig. 8D). The above results were verified through qRT-PCR and western blotting assay (Fig. 8E–G). Taken together, our results prove that Gankyrin knockdown inhibits NSCLC tumorigenesis.

## Discussion

This study determined the important role of Gankyrin in accelerating NSCLC growth through a combination of human data with cellular and mouse experiments. Gankyrin expression is higher in the tumor tissues of NSCLC patients than in adjacent tissues. The results of in vivo and in vitro experiments indicated that silencing Gankyrin suppresses tumorigenesis in a glycolysis-dependent manner. Mechanistically, Gankyrin leads to the nuclear translocation of YAP1 and promotes the transcription and translation of glycolysis-related genes. Our results are consistent with those of previous studies that have reported Gankyrin as an essential regulator of NSCLC progression and uncovered its role in NSCLC metabolism.

Gankyrin functions as an oncogene in several human cancers, including hepatocellular carcinoma [11], renal cell carcinoma [17], and gastric cancer [18]. Previous studies have focused on the protein–protein interactions and proteasomal degradation that occur as functions of Gankyrin in carcinogenesis [19–22]. Gankyrin also plays a metabolic reprogramming role in the regulation of hepatocarcinogenesis, metastasis, and drug resistance [16]. Yang et al. have reported that Gankyrin is involved in the production of ROS and suggested a link between oxidative stress and HCC development [20]. These findings suggest that Gankyrin an oncoprotein, has different functions in various tumor. However, the molecular mechanism of action of Gankyrin in lung cancer metabolism remains unclear. Here, we identified a Gankyrin-dependent regulatory network involving YAP1 and glycolysis that controls metabolism during NSCLC initiation. We found that Gankyrin was overexpressed in a variety of NSCLC cell lines compared to BEAS-2B, suggesting that it regulates certain biological processes that participate in NSCLC tumorigenesis. Importantly, we found that Gankyrin silencing inhibited the proliferation, migration, invasion, and EMT in NSCLC cells. Thus, inhibiting Gankyrin activity may be an effective anticancer strategy.

Metabolic disorders are emerging as a hallmark of cancer, with increased glucose uptake and lactic acid production increasingly suspected of supporting the malignant biological processes associated with cancer [23]. Accumulating evidence indicates that NSCLC cells exhibit extensive metabolic heterogeneity and engage differentially in glycolysis and mitochondrial oxidation [24, 25]. PKM2 promotes glycolysis and the Warburg effect while inhibiting the mitochondrial respiratory pathway by negatively regulating H2Bub1 [26]. Blocking of PGAM1 suppresses mTOR-mediated tumor growth, and PGAM1 abundance is an unfavorable predictor in patient survival [27]. Additionally, the key glycolytic enzyme ENO1 activates the FAK/PI3K/AKT pathway to regulate glycolysis, the cell cycle, and EMT-associated genes [28]. Therefore, searching for metabolism-related genes may facilitate the identification of mechanisms that regulate the pathophysiological process of NSCLC. In this study, we discovered that the oncogene Gankyrin participates in NSCLC cell metabolism, especially glycolysis. Gankyrin has also been reported to activate β-catenin/c-Myc signaling, enhancing glycolysis and glutaminolysis, thereby promoting hepatocarcinogenesis and drug resistance [16]. Our experiment demonstrated that Gankyrin, which is upregulated in NSCLC, facilitates glycolysis by promoting YAP1 expression. Furthermore, silencing Gankyrin was found to inhibit expression of the key glycolytic enzymes HK2, PKM2, PGK1, and LDHA at the transcriptional and protein levels while enhancing lactic acid and ATP production. In addition, Gankyrin robustly increased the glucose uptake and consumption [29, 30]. Surprisingly, Gankyrin was found to increase the ECAR but did not affect the OCR. This finding may be attributed to the fact that the overexpression of Gankyrin activates the intrinsic activity of mitochondria but reduces their number, therefore, the overall OCR value did not change significantly. Further accurate detection of the various changes that occur inside the mitochondria is required via subsequent experiments to accurately elucidate this process.

Growing evidence suggests that the Hippo pathway is an evolutionarily conserved signaling cascade that regulates multiple biological processes, including organ size control, tissue regeneration, immune modulation, tumorigenesis, and stem cell functions. The Hippo signaling pathway is a highly conserved tumor suppressor pathway, mainly including Ste20-like kinase 1 (MST1) and large tumor suppressor 1 (LATS1), Yes associated protein 1 (YAP1) and its analog TAZ, and the regulatory subunits of MST1 and LATS1, MOB1 and SAV1. MST1 and LATS1 are two types of tumor suppressor kinases [31]. When the Hippo pathway is inactivated, the activities of MST1 and LATS1 are inhibited; hence YAP1 can neither be phosphorylated nor transferred to the nucleus to bind to the TEAD transcription factor. It promotes the expression of downstream target genes, such as *CTGF* and *CYR61*, which are related to cell growth, proliferation and survival [32]. In this study, we found that Gankyrin overexpression did not affect MST1 and LATS1 expression, or their phosphorylation. This result indicates that Gankyrin does not affect these suppressors upstream of the Hippo signaling pathway. Excessive activation of the downstream effector YAP1 contributes to cancer development [33]. Recently, Jin et al. have suggested that YAP1 inhibits autophagy and promotes the progression of colorectal cancer by upregulating Bcl-2 expression [34]. YAP1 also governs epigenetic regulatory networks and dictates metabolic homeostasis via Myc, Sox2, and p53 in pancreatic ductal adenocarcinomas [35]. In our previous study, we found that YAP1 promoted NSCLC cell invasion and migration by regulating Slug transcription via the transcription co-factor TEAD [15]. In this study, Gankyrin was not found to affect the expression of other members of the upstream Hippo pathway but only acted on YAP1 by increasing its expression, inhibiting its phosphorylation, and promoting its nuclear translocation. Gankyrin was also found to promote the proliferation of NSCLC cells, inhibit apoptosis, and accelerate the EMT process via YAP1 activation. According to a recent study YAP1 stimulates nucleotide biosynthesis by reprogramming nitrogen metabolism, and participates in both development and tumorigenesis [36]. YAP1 has also been shown to be regulated by metabolic pathways, such as aerobic glycolysis, mevalonate synthesis, and glutaminolysis during the development of tumor [37, 38]. Here, we found that YAP1 frequently increased glucose uptake and lactic acid production in A549 and H460 cells. Interestingly, silencing YAP1 inhibited the increase in ECAR caused by Gankyrin. This result indicates that Gankyrin regulates the cellular glycolytic process mediated by YAP1. Our data provide evidence for the YAP1-mediated downstream regulation of Gankyrin in NSCLC. Several studies have demonstrated that Gankyrin can also act as a nuclear-cytoplasmic shuttling protein that exports NF-κB from the nucleus [39, 40]. This finding may explain why Gankyrin promotes entry of YAP1 into the nucleus. However, further investigation is required to elucidate how Gankyrin regulates the subcellular localization of YAP1.

In conclusion, our results indicate that the binding of Gankyrin to YAP1 regulates tumorigenesis and metastasis in NSCLC, implying that the interaction between Gankyrin and YAP1 is associated with energy metabolism. Our results illustrate that the interaction between Gankyrin and YAP1, which regulated glycolysis in tumor cells, may be a novel opportunity for the treatment of NSCLC in the future.

## Materials and methods

### Human tissue samples

Human lung cancer tissues and paired adjacent lung tissues were obtained from the Second Affiliated Hospital of Harbin Medical University between August 2019 and May 2021. All included patients were diagnosed with NSCLC and received no neoadjuvant radiotherapy or chemotherapy before surgery. This study was approved by the Research Ethics Committee at the Second Affiliated Hospital of Harbin Medical University (KY2019-139).

### Cell culture

Normal human bronchial epithelium transformed with Ad12-SV40 2B (BEAS-2B) and the NSCLC cell lines A549, H460, H1299, H1650, and H1975 were purchased from the Cell Bank of the Chinese Academy of Sciences (Shanghai, China). BEAS-2B was cultured in DMEM (VivaCell, Shanghai, China). H460, H1299, H1650, and H1975 cells were grown in RPMI 1640 medium (VivaCell, Shanghai, China). A549 cells were cultured in an F-12K mixture nutrient (Gibco, Grand Island, NY, USA). The stable Gankyrin-knockdown cell lines A549-sh-Gankyrin and A549-sh-NC were purchased from Hanbio Biotechnology (Shanghai, China). Cells were cultured with 10% fetal bovine serum (FBS, Biological Industries, Kibbutz Beit-Haemek, Israel) and 1% penicillin and streptomycin (Solarbio, Beijing, China) under 5% CO_2_ at 37 °C, authenticated by short tandem repeat profiling and tested free from mycoplasma.

### Plasmid construction and small interfering RNA (siRNA) reagent transfection

pGV219-Gankyrin and pGV219-YAP1 plasmids were synthesized by the GeneChem Corporation (Shanghai, China) and transfected using Lipofectamine 2000 (Invitrogen, Carlsbad, USA) according to the manufacturer’s instructions. The pGV219 vector contains CMV-MCS-SV40-Neomycin elements. An empty vector was used as a negative control. YAP1 and Gankyrin siRNAs were synthesized by Sigma (Sigma-Aldrich, St. Louis, MO, USA) and RiboBio Corporation (Guangzhou, China), respectively.

### Quantitative real-time PCR (qRT-PCR)

Total RNA from cells or tissues was isolated using TRIzol reagent (Invitrogen, Carlsbad, CA, USA) according to the manufacturer’s instructions. cDNAs were generated using All-in-one First-strand cDNA Synthesis SuperMix (Transgene biotech, Beijing, China). qRT-PCR was performed by 7500 Fast Real-Time PCR System (Applied Biosystems, ABI, USA) for 40 cycles using SYBR Green qPCR SuperMix (Transgene biotech, Beijing, China). The relative mRNA expression levels were calculated based on Ct values and normalized to *ACTB* levels to create internal controls for each sample using the 2^-△△Ct^ method. Sequences of primers were listed in Supplementary Table S1.

### Western blotting analysis

Total protein samples were extracted from NSCLC cells and tumor tissues collected from human or nude mice using RIPA lysis buffer (Beyotime, Jiangsu, China, P0013K) and protease inhibitor cocktail (Roche, Germany, 04963159001) at a ratio of 100:1. Electrophoretic experiments were performed with an equal amount of protein, and the samples were transferred to the nitrocellulose blotting membrane (Merck Millipore, R7BA46025). The membrane was blocked with the Western Quick Block Kit (NCM Biotech, Suzhou, China) for 10 min and probed with primary antibodies against fibronectin 1 (FN1, Proteintech, Rosemont, IL, USA, 15613-1-AP 1:1000), ZO-1 (Proteintech, 21773-1-AP, 1:500), E-cadherin (Proteintech, 20874-1-AP, 1:500), YAP1 (Proteintech, 13584-1-AP, 1:1000), Vimentin (Cell Signaling, #5741, 1:1000), Gankyrin (Abcam, Cambridge, UK, ab182576, 1:1000) and β-actin (Proteintech, 66009-1-Ig, 1:10,000) overnight at 4 °C. The membranes were then incubated with anti-mouse or anti-rabbit secondary antibodies (Abcam, Cambridge, UK, 1:10,000) for 50 min at room temperature. Finally, immunoreactivity was measured using the Odyssey Infrared Imaging System (Odyssey, LICOR, USA).

### Co-immunoprecipitation

The cells were lysed with a lysis buffer containing a protease inhibitor cocktail. The primary antibody or control IgG was diluted with binding buffer to a final concentration of 50 μg/ml, mixed with cleaned protein A/G magnetic beads (Med Chem Express, Shanghai, China) and incubated under rotation for 2 h at 4 °C. The magnetic bead-antibody complex was then washed with washing buffer and cell lysate was added. The resulting mixture was rotated overnight at 4 °C to allow antigen binding to the magnetic bead-antibody complex. Finally, the complex was boiled in loading buffer for 5 min at 95 °C, to produce final solution for SDS-PAGE.

### MTT assay

Cells were seeded in a 96-well plate, attached overnight, then transfected according to the designed. After 48 h, 20 μl MTT (M21828, Sigma, USA) and 180 μl RPMI 1640 were added to each well before incubating the placed at 37 °C and 5% CO_2_ for 4 h in the dark. Formazan was dissolved in 150 μl DMSO and rocked for 15 min at room temperature. Finally, the absorbance of the culture solution was measured at 490 nm using an Infinite®200PRO microplate spectrophotometer (Tecan, Salzburg, Austria).

### Wound-healing assay

Cell migration was measured using a wound-healing assay determined, as described previously [15]. Cells were inoculated into 6-well plates and cultured overnight in 10% FBS prior to wound preparation, allowing the cells to multiply to a density of approximately 90%-100%. Sterile 10-μl micropipette tips were then used to produce a scratch wound in the cell monolayers, and the cells were cultured in serum-free medium to inhibit cell proliferation in the wound-healing assay. Images of wound healing at different stages were collected using a microscope 0, 24, and 48 h after cell transfection.

### Transwell assay

To detect invasion and migration, cells were cultured in Transwell plate chambers (#3422, Corning, NY, USA) with or without Matrigel mixture (BD Biosciences San Jose, CA). Culture medium containing 10% FBS was added to the lower chambers but not to the upper chambers. Cells capable of migration and invasion were induced to transfer laterally through the compartment membrane. The cells were then stained with 0.5% crystal violet and counted under a microscope (Olympus, Tokyo, Japan).

### Immunofluorescence staining and confocal microscopy

Cells were cultured on a microscope cover glass that was placed at the bottom of a 24-well plate and transfected as described previously. The cells were then left for 48 h, fixed with 4% paraformaldehyde for 30 min at room temperature, and permeabilized for 1 h. After blocking with goat serum (Boster, California, USA) for 45 min, cells were incubated with primary antibodies overnight at 4 °C and incubated with Alexa Fluor 488-tagged or 594-tagged secondary antibodies (Proteintech, USA) at 1:200 dilution for 1 h at room temperature. Nuclei were stained with DAPI (Beyotime, Jiangsu, China) for 10 min. Images were collected using a Zeiss confocal laser-scanning microscope (Carl Zeiss, Oberkochen, Germany).

### Immunohistochemistry (IHC) analysis

Samples collected from human patients and nude mouse model tumor tissue sections were de-paraffinized in xylene and rehydrated with gradient ethanol. After washing with double-distilled water, the tissue sections were incubated with 3% hydrogen peroxide (Solarbio, Beijing, China) for 10 min and antigenic repair was performed with sodium citrate antigen retrieval solution (Solarbio, Beijing, China). The sections were then blocked with 50% goat serum for 1 h and incubated with primary antibodies anti-E-cadherin (Proteintech, USA, 1:100), anti-Vimentin (Cell Signaling, USA, 1:200), anti-Gankyrin (Abcam, UK, 1:200) or anti-YAP1 (Proteintech, USA, 1:200) overnight at 4 °C before incubation with a horseradish peroxidase (HRP)-conjugated secondary antibody (Zhongshan Technology, Beijing, China) for 30 min at 37 °C. The tissue sections were stained with DAB (Zhongshan Technology) staining solution and counterstained with hematoxylin (LABEST, Beijing, China) for 10 s. Photographs were obtained using a Zeiss microscope (Carl Zeiss, Oberkochen, Germany).

### Measurement of ATP content

The cells were cultured in 6-well plates and transfected as required. ATP content was measured using an ATP assay kit (Beyotime, Jiangsu, China), according to the manufacturer’s instructions. The results were obtained based on the standard curve.

### Measurement of lactic acid production

The Lactic acid production was detected using Lactic Acid Assay Kit (Solarbio, Beijing, China, BC2230), according to the manufacturer’s instructions. The results were obtained based on the standard curve.

### Measurement of glucose consumption

Concentrations of glucose in the cells were determined using glucose assay kit (Solarbio, Beijing, China, BC2500). In brief, supernatant was collected, and the system was prepared according to the manufacturer’s instructions. Finally, the absorbance, which represented glucose content, was measured at 505 nm.

### Extracellular acidification rate (ECAR) and oxygen consumption rate (OCR) measurement

The Seahorse Extracellular Flux Analyzer XF24 (Seahorse Bioscience, Seahorse Biosciences, Billerica, MA, USA) was used in accordance with the manufacturer’s instructions to monitor cellular glycolysis and respiration rates in vitro. A549 cells were seeded in an XF24-well plate and incubated overnight, followed by serum starvation for 24 h. The cells were then incubated with non-buffered medium and injected with 10 mM glucose, 1 µM oligomycin, and 80 mM 2-deoxyglucose sequentially to detect the real-time ECAR (in mpH/min). The OCR (in pmol/min) was analyzed using the Cell Mito Stress Test kit (Agilent Technologies) using oligomycin, FCCP, and rotenone.

### Xenograft model of lung cancer

Five-week-old male BALB/c nude mice were purchased for the xenograft studies from Beijing Vital River Laboratory Animal Technology. For the xenograft mouse model, 1 × 10^6^ cells containing A549-sh-Gankyrin or A549-sh-NC were dissolved in 100 μl PBS and implanted subcutaneously into the left armpit. The volume of the tumor was measured using a vernier caliper every four days. After four weeks, all mice were sacrificed, and the tumor weight was measured using an analytical balance. Animals were randomly assigned to experimental groups. Researchers were blinded to the group allocation both during the experiment when assessing the outcome. Sample sizes is ≥3 were used for each condition. All experiments were performed in accordance with the guiding principles for the care and use of laboratory animals at The Second Affiliated Hospital of Harbin Medical University and approved by the Ethics Committee for Animal Experiments (sydwgzr2021-207).

### Statistical analysis

Gene expression cohorts of NSCLC with normal adjacent tissue samples were collected from TCGA database which were downloaded from Genomic Data Commons (https://portal.gdc.cancer.gov). The log2-transformed expression levels were used to preprocess TCGA mRNA expression profiles. Pearson’s correlation was used to analyze the correlation between *PSMD10* expression and glycolysis genes, with *r* > 0.2 considered a significant correlation. All analytical processes were performed using the R software version 4.0.1. All data are expressed as mean ± SEM. The unpaired Student’s *t*-test was used for two-group comparisons. One-way ANOVA was used for multiple groups, followed by Dunnett’s test for multiple comparisons. In all analyses, statistical significance was assumed at less than 5% (*P* < 0.05). Statistical analyses were performed using GraphPad Prism 8. No estimate of variation was performed within any dataset prior to statistical analysis. The variance was similar in the comparison groups. All experiments were performed at least three independent times, and similar results were obtained each time.

## Supplementary information

Original Data File

Supplementary material

Clinical sample Ethics statement

Animal Ethics statement

Editing Certificate

## Data Availability

All data that support the conclusions of this study are available from the corresponding author upon reasonable request.
