# Supplementary material for: Gankyrin modulated non-small cell lung cancer progression via glycolysis metabolism in a YAP1-dependent manner
Source: Cell Death Discov. 2022 Jul 9;8:312. doi: 10.1038/s41420-022-01104-3 (PMC9271063; doi:10.1038/s41420-022-01104-3)

Figure 1D

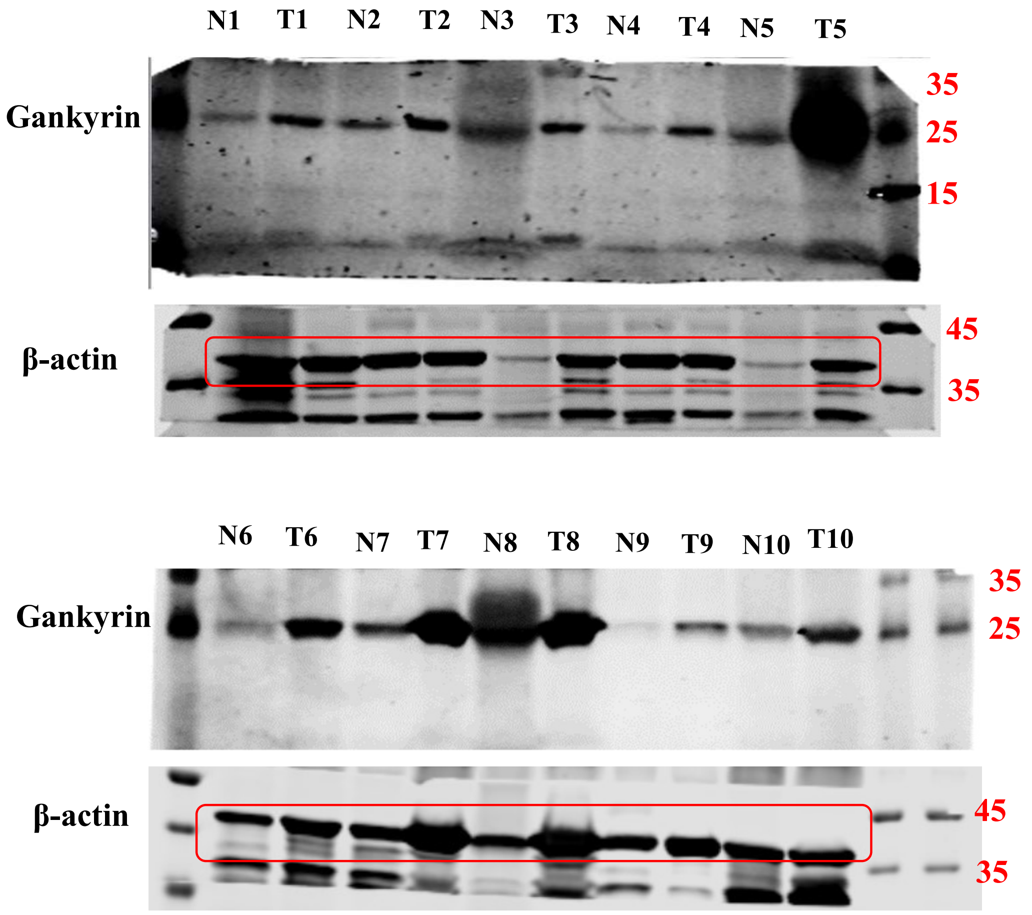

Figure 1F

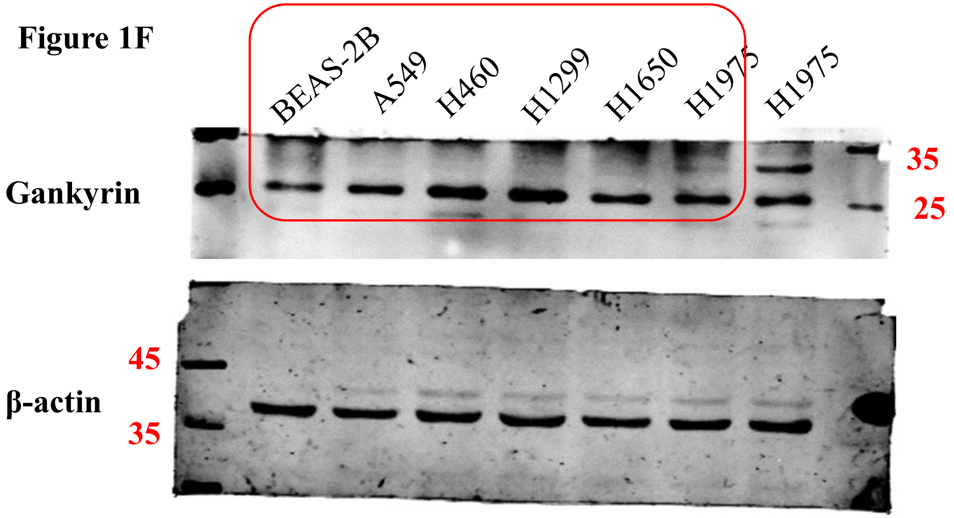

Figure 2G

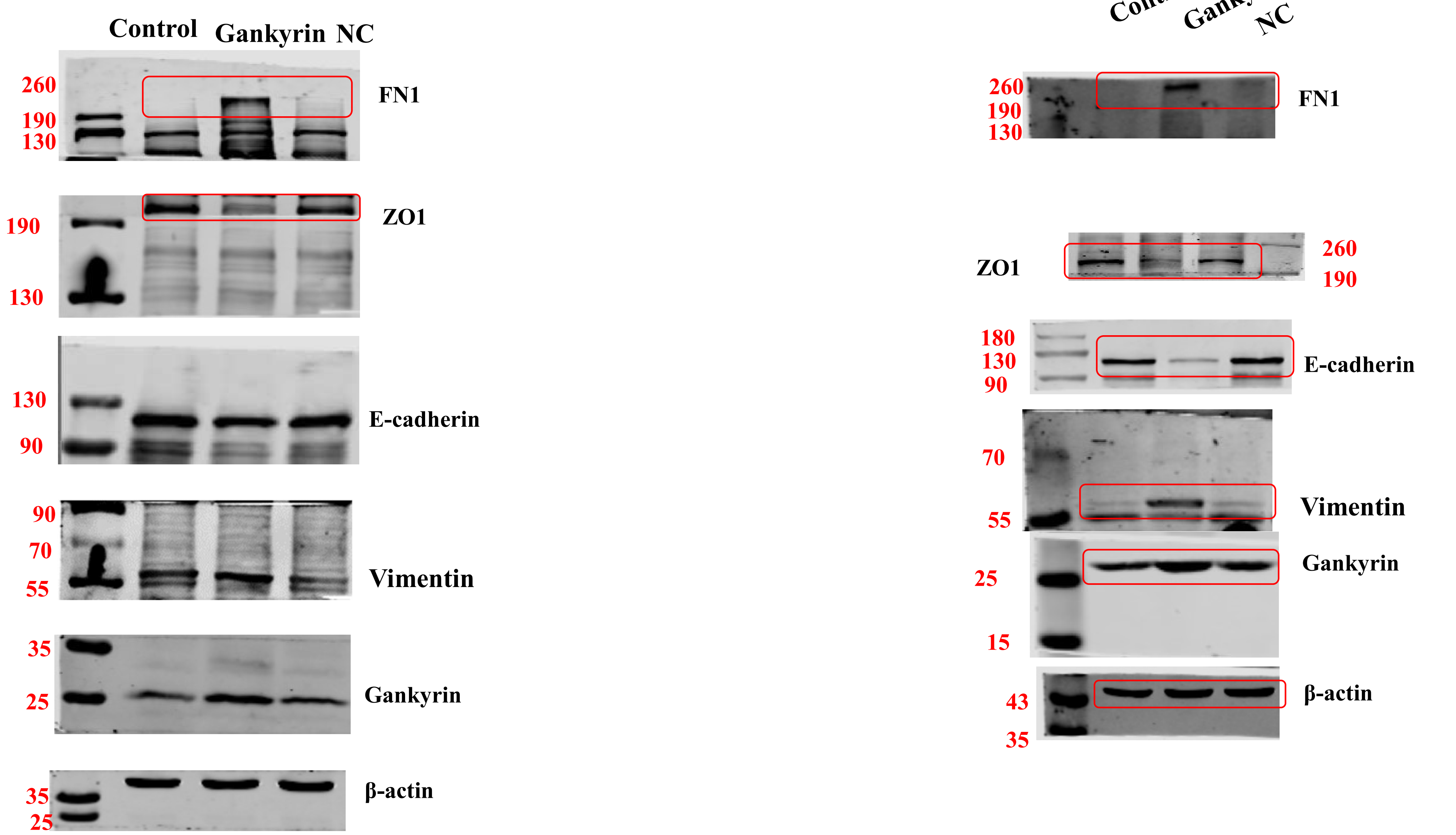
A549
Control
Gankyrin
NC
260
190
130
FN1
190
130
ZO1
130
90
E-cadherin
90
70
55
Vimentin
35
25
Gankyrin
35
25
β-actin
H460
Control
Gankyrin
NC
260
190
130
FN1
ZO1
260
190
180
130
90
E-cadherin
70
55
Vimentin
25
15
Gankyrin
43
35
β-actin

Figure 3G

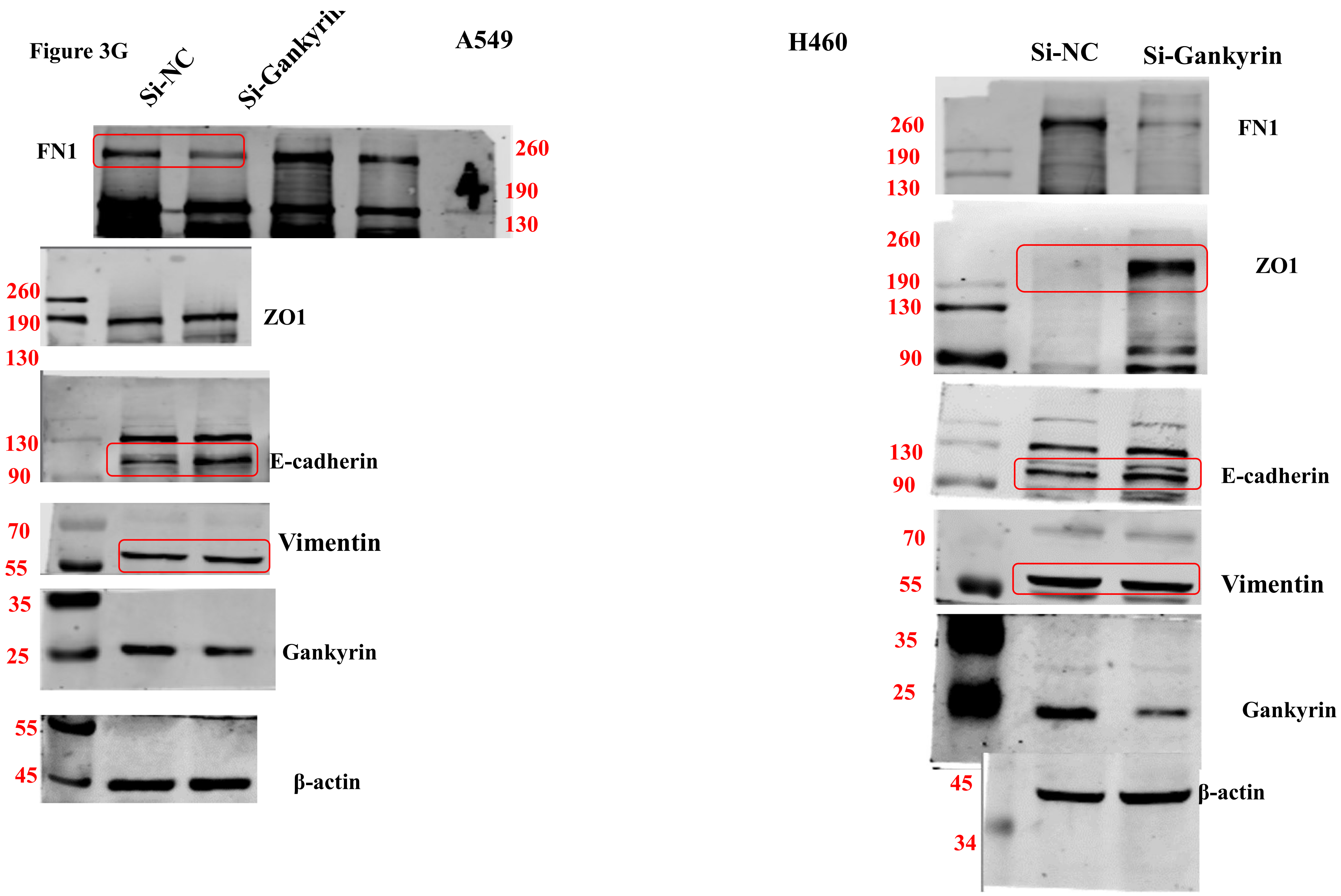

A549
Si-NC
Si-Gankyrin
FN1
260
190
130
ZO1
E-cadherin
90
Vimentin
70
55
Gankyrin
35
25
β-actin
45
H460
34

Figure 4C

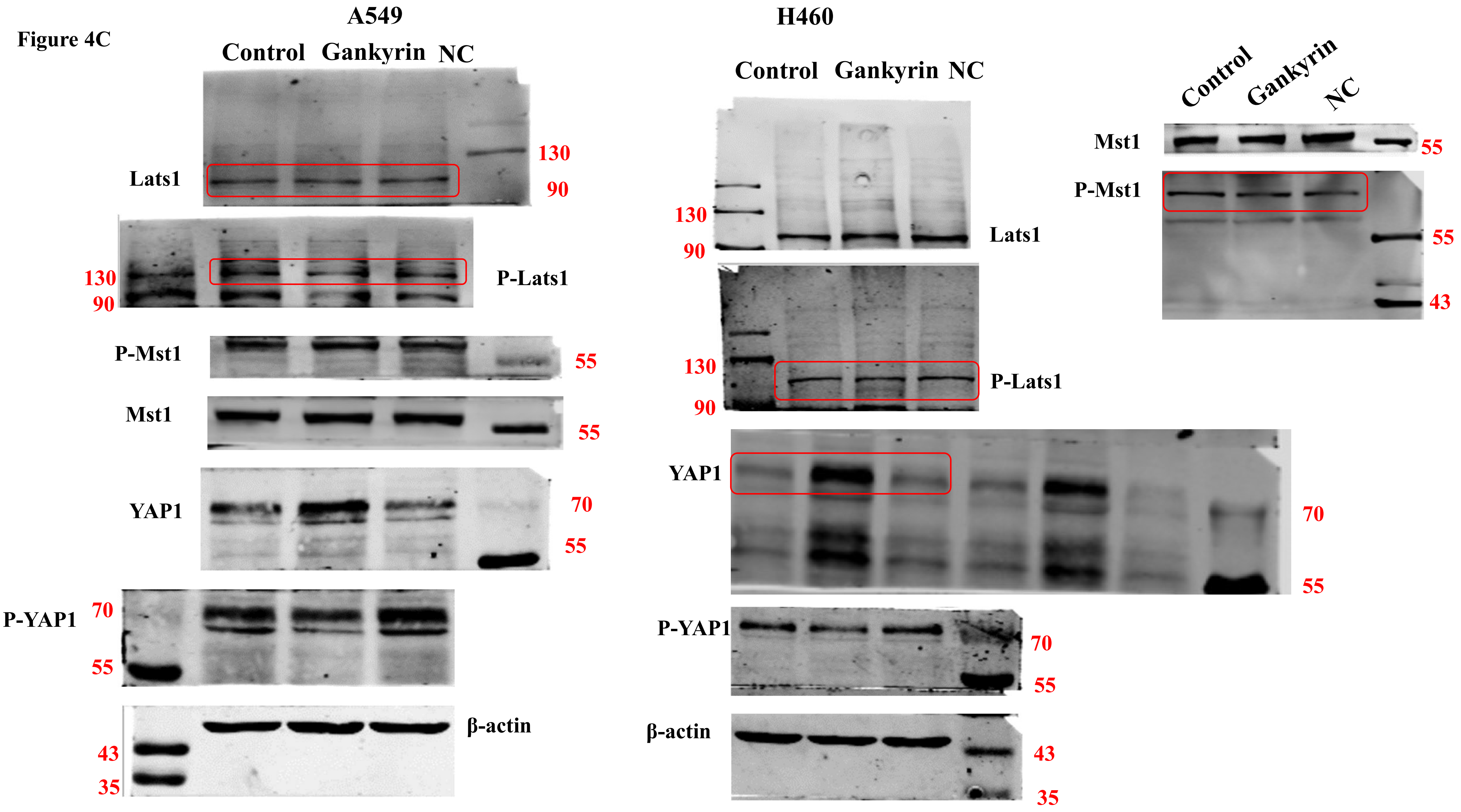

A549
Control Gankyrin NC
Lats1
130
90
P-Lats1
130
90
P-Mst1
55
Mst1
55
YAP1
70
55
P-YAP1
70
55
β-actin
43
35
H460
Control Gankyrin NC
Lats1
130
90
P-Lats1
130
90
YAP1
70
55
P-YAP1
70
55
β-actin
43
35
Control
Gankyrin
NC
Mst1
55
P-Mst1
55
43

Figure 4D

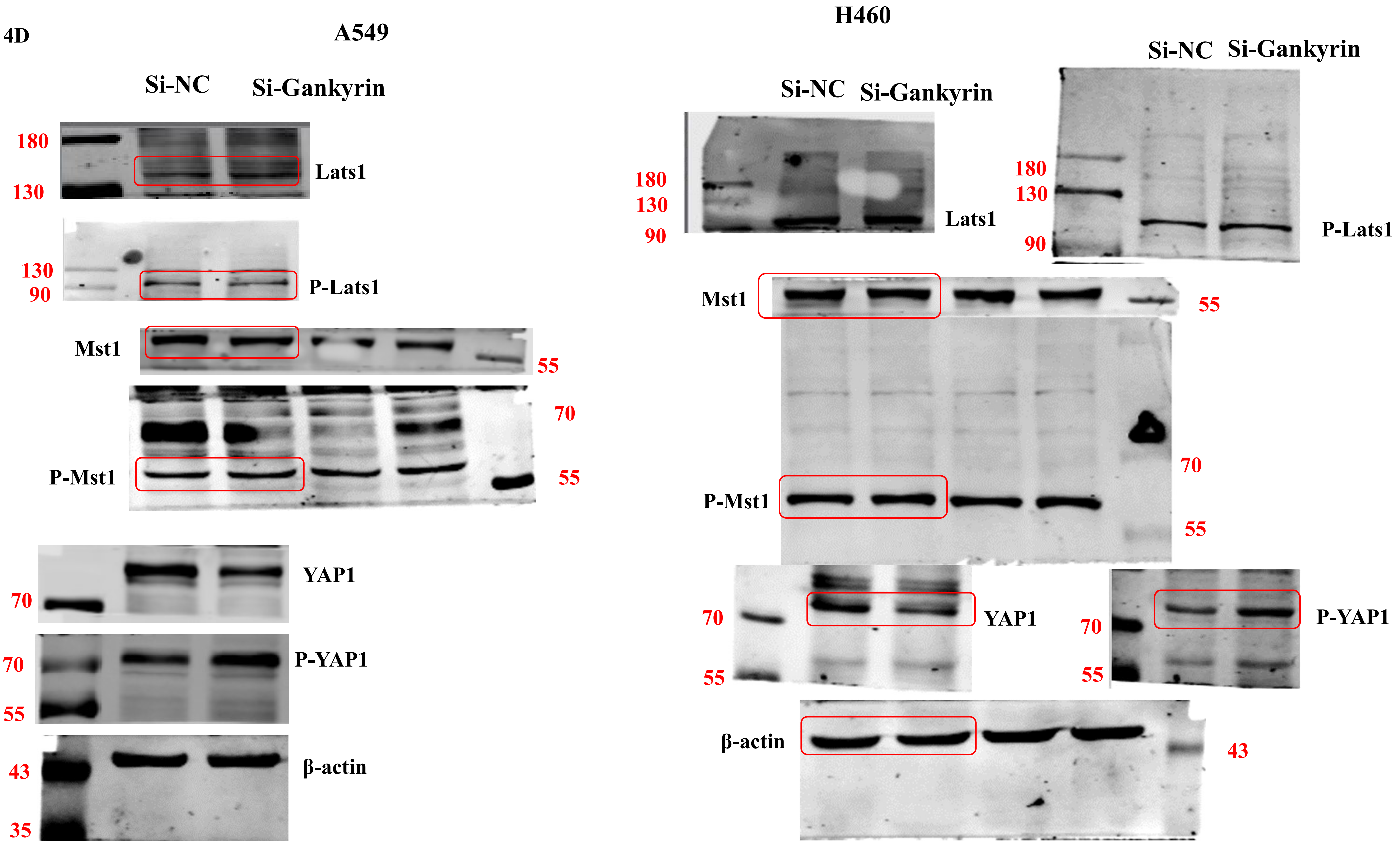
A549
Si-NC
Si-Gankyrin
180
130
Lats1
130
90
P-Lats1
Mst1
55
70
P-Mst1
55
YAP1
70
70
P-YAP1
55
43
β-actin
35
H460
Si-NC
Si-Gankyrin
180
130
90
Lats1
Si-NC
Si-Gankyrin
180
130
90
P-Lats1
Mst1
55
70
P-Mst1
55
70
YAP1
55
70
P-YAP1
55
β-actin
43

Figure 4F

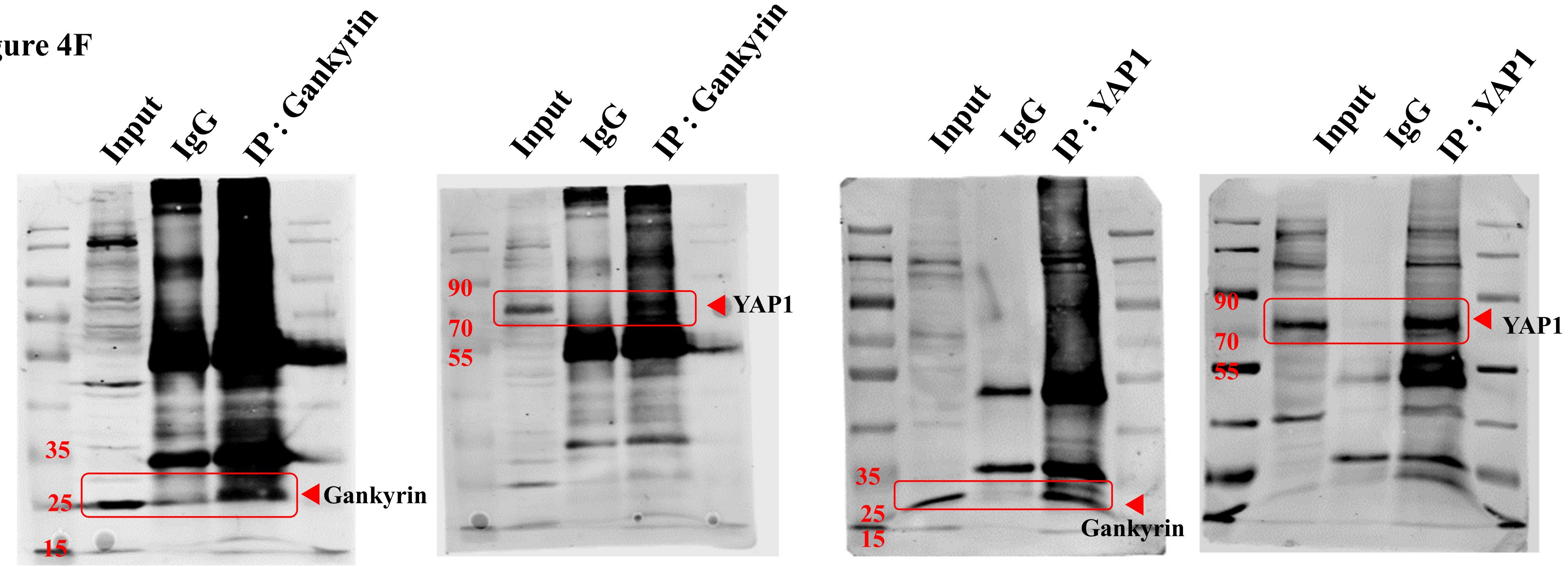

Input
IgG
IP : Gankyrin
35
25
15
Gankyrin
Input
IgG
IP : Gankyrin
90
70
55
YAP1
Input
IgG
IP : YAP1
35
25
15
Gankyrin
Input
IgG
IP : YAP1
90
70
55
YAP1

Figure 4F

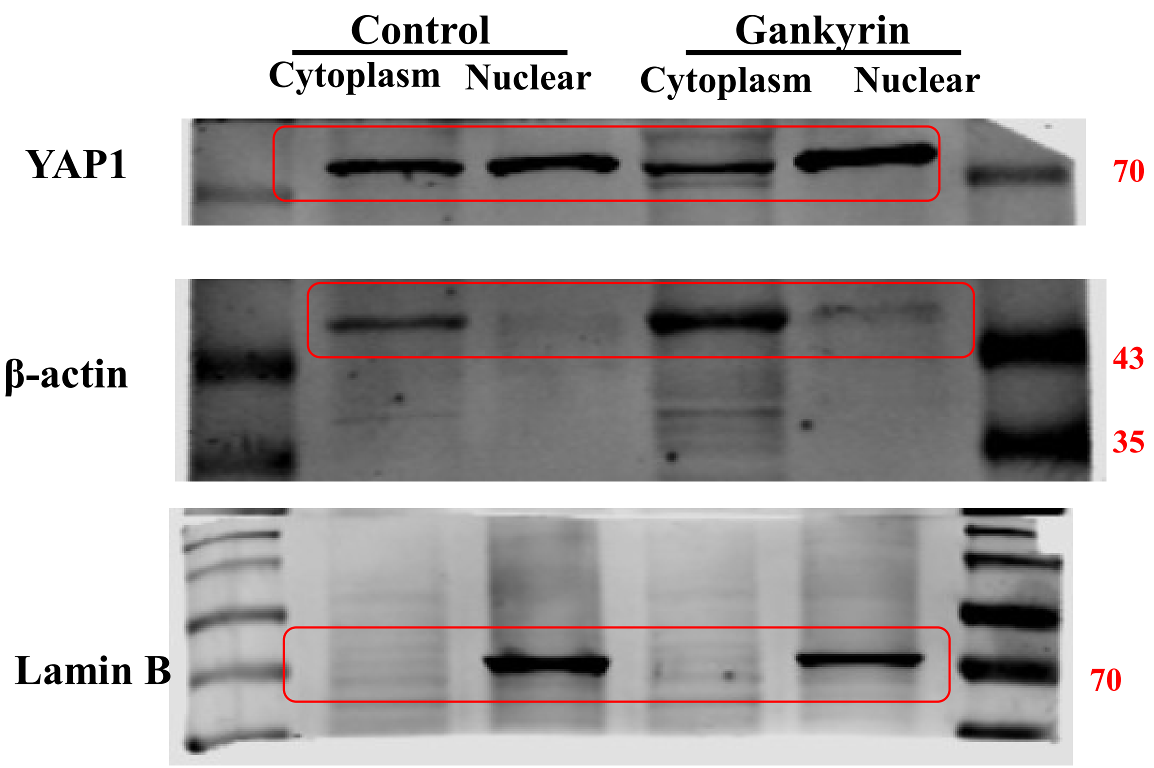
Control
Gankyrin
Cytoplasm
Nuclear
Cytoplasm
Nuclear
YAP1
70
β-actin
43
35
Lamin B
70

Figure 5G

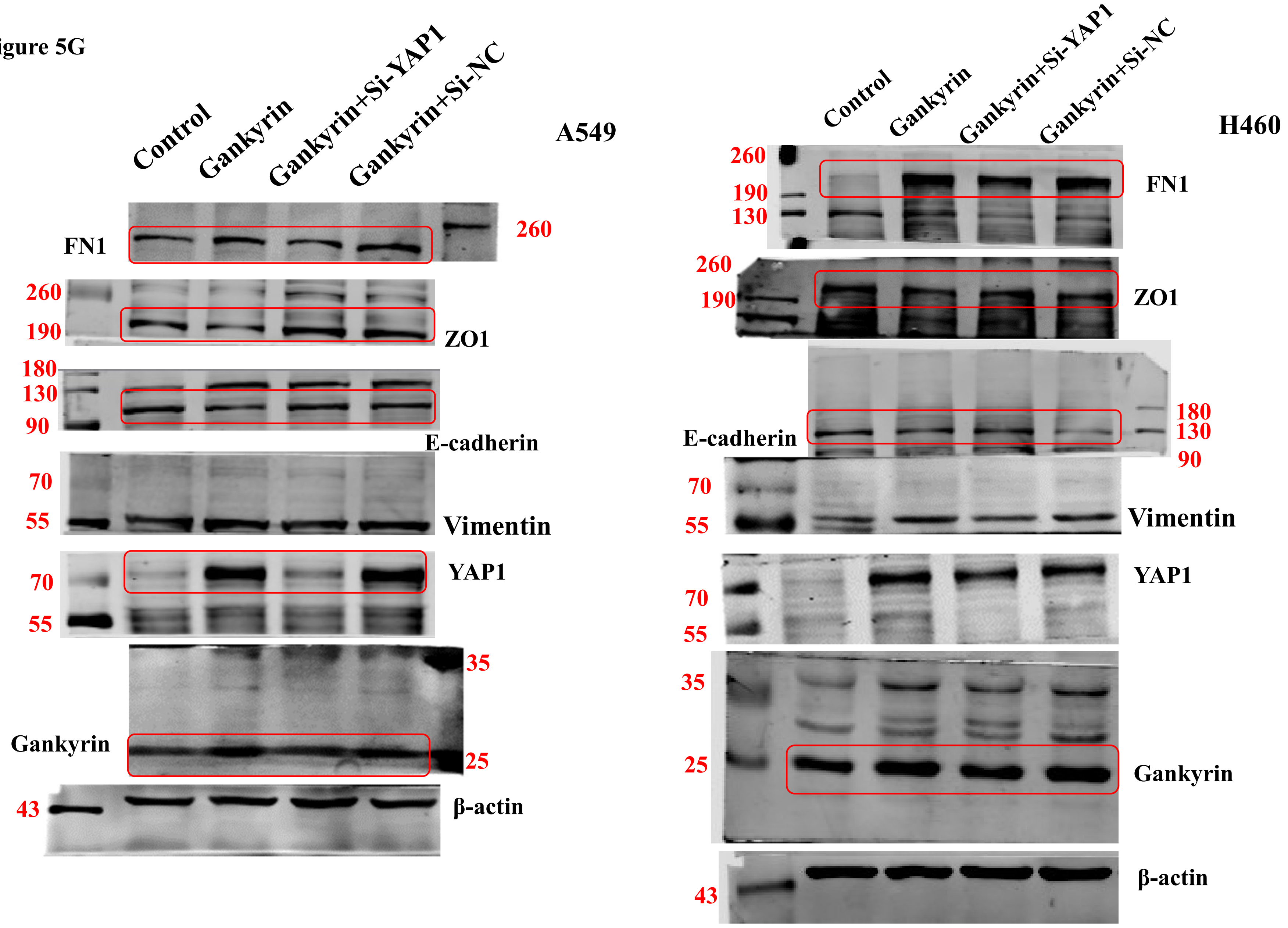

Control
Gankyrin
Gankyrin+Si-YAP1
Gankyrin+Si-NC
A549
FN1
260
260
190
ZO1
180
130
90
E-cadherin
70
55
Vimentin
70
55
YAP1
35
Gankyrin
25
43
β-actin
Control
Gankyrin
Gankyrin+Si-YAP1
Gankyrin+Si-NC
H460
260
190
130
FN1
260
190
ZO1
E-cadherin
180
130
90
70
55
Vimentin
YAP1
70
55
35
25
Gankyrin
43
β-actin

Figure 7D

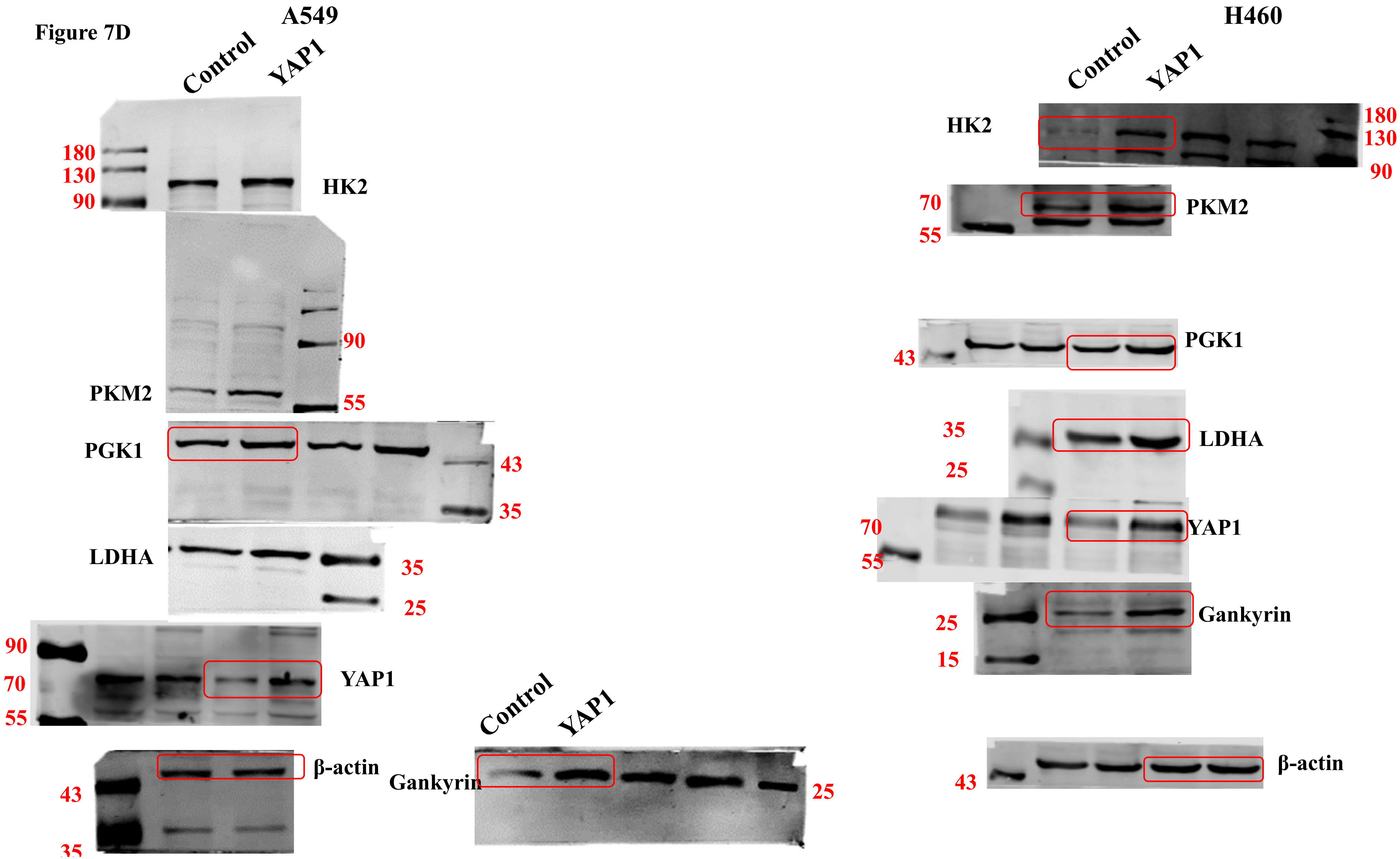
A549
Control
YAP1
180
130
90
HK2
90
PKM2
55
PGK1
43
35
LDHA
35
25
90
70
55
YAP1
β-actin
43
35
Control
YAP1
Gankyrin
25
H460
Control
YAP1
HK2
180
130
90
70
PKM2
55
43
PGK1
35
LDHA
25
70
YAP1
55
25
Gankyrin
15
43
β-actin

Figure 7E

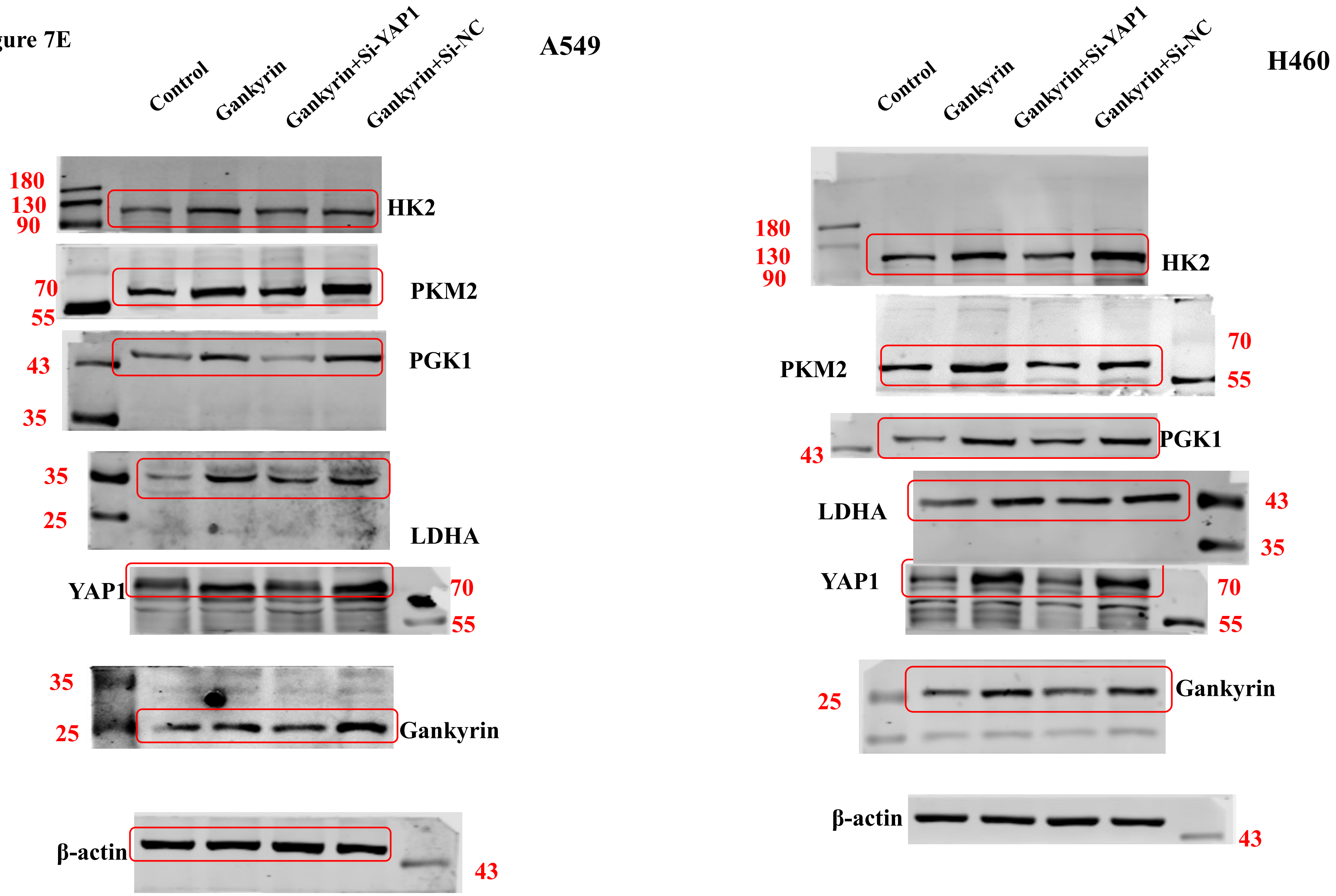
A549
Control
Gankyrin
Gankyrin+Si-YAP1
Gankyrin+Si-NC
180
130
90
HK2
70
55
PKM2
43
35
PGK1
35
25
LDHA
YAP1
70
55
35
25
Gankyrin
β-actin
43
H460
Control
Gankyrin
Gankyrin+Si-YAP1
Gankyrin+Si-NC
180
130
90
HK2
PKM2
70
55
43
PGK1
LDHA
43
35
YAP1
70
55
25
Gankyrin
β-actin
43

Figure 8G

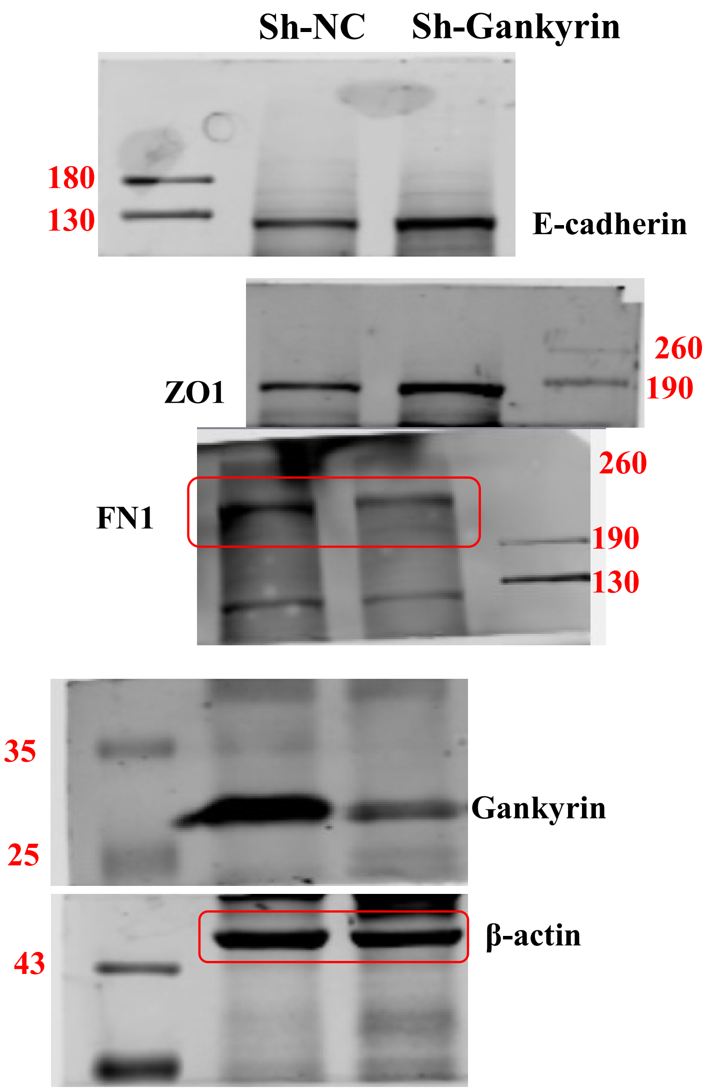

Sh-NC
Sh-Gankyrin
180
130
E-cadherin
ZO1
260
190
FN1
260
190
130
35
Gankyrin
25
43
β-actin

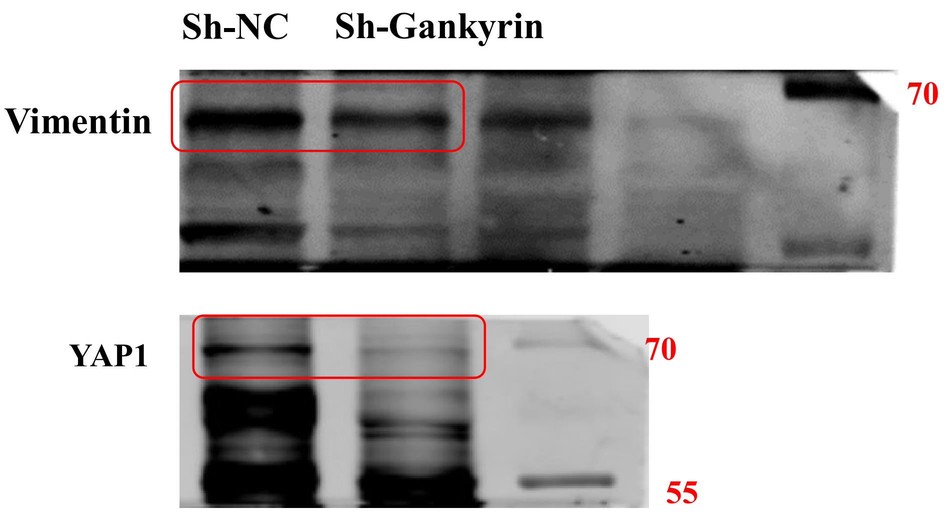

Sh-NC
Sh-Gankyrin
Vimentin
70
YAP1
70
55

Supplementary figure S2 F A549

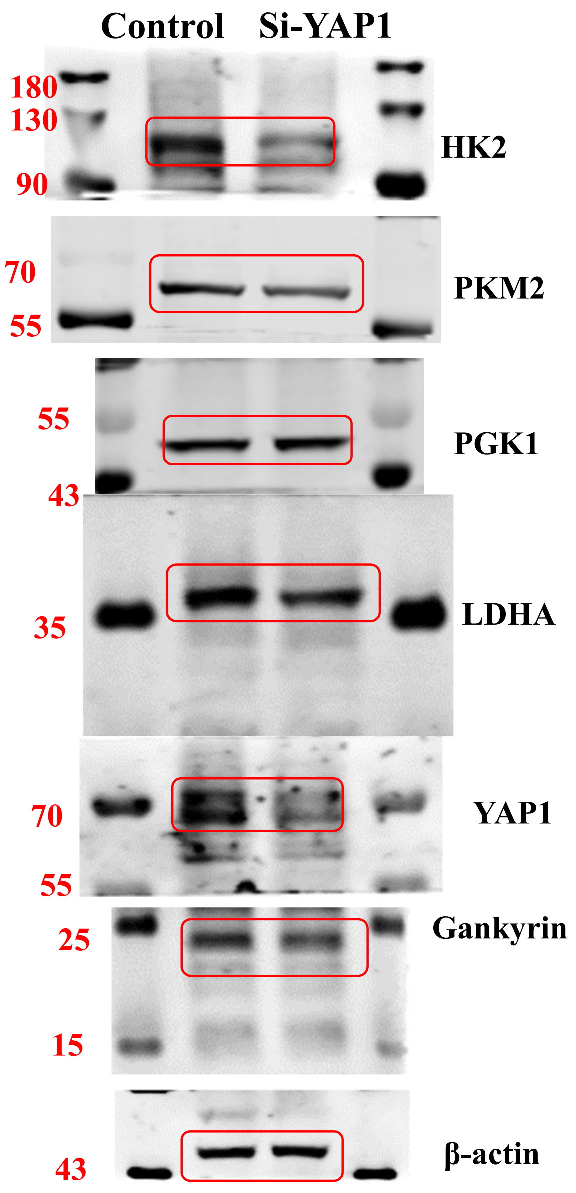

Supplementary figure S2 G H460

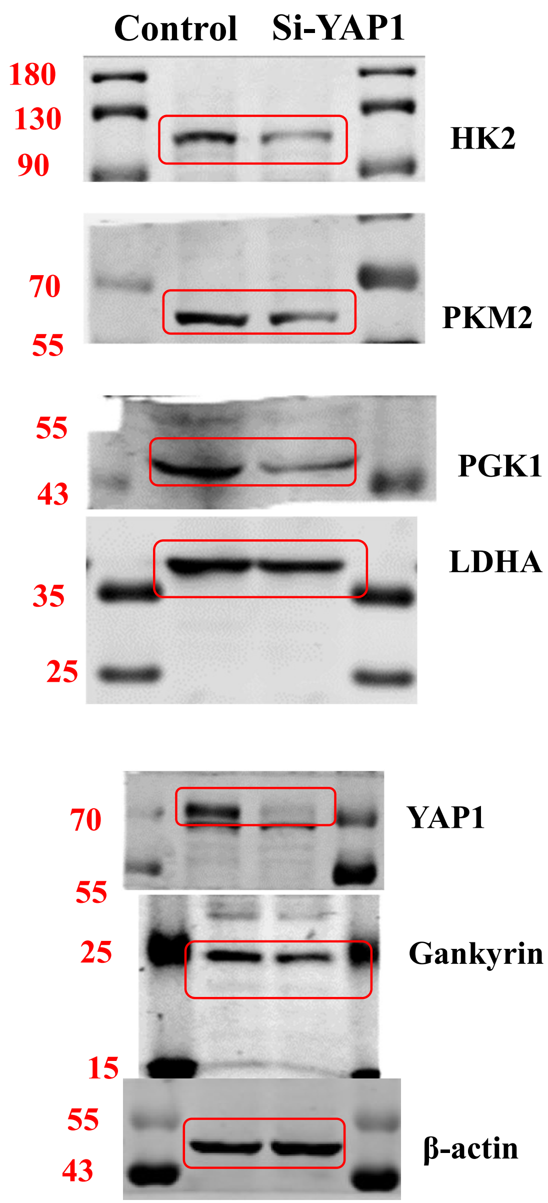

Supplement: Supplementary file 1 — Original Data File [file 41420_2022_1104_MOESM1_ESM.pdf]
