# Supplementary material for: Gankyrin modulated non-small cell lung cancer progression via glycolysis metabolism in a YAP1-dependent manner
Source: Cell Death Discov. 2022 Jul 9;8:312. doi: 10.1038/s41420-022-01104-3 (PMC9271063; doi:10.1038/s41420-022-01104-3)
Supplement: Supplementary file 2 — Supplementary material [file 41420_2022_1104_MOESM2_ESM.docx]

**Supplementary figure S1**


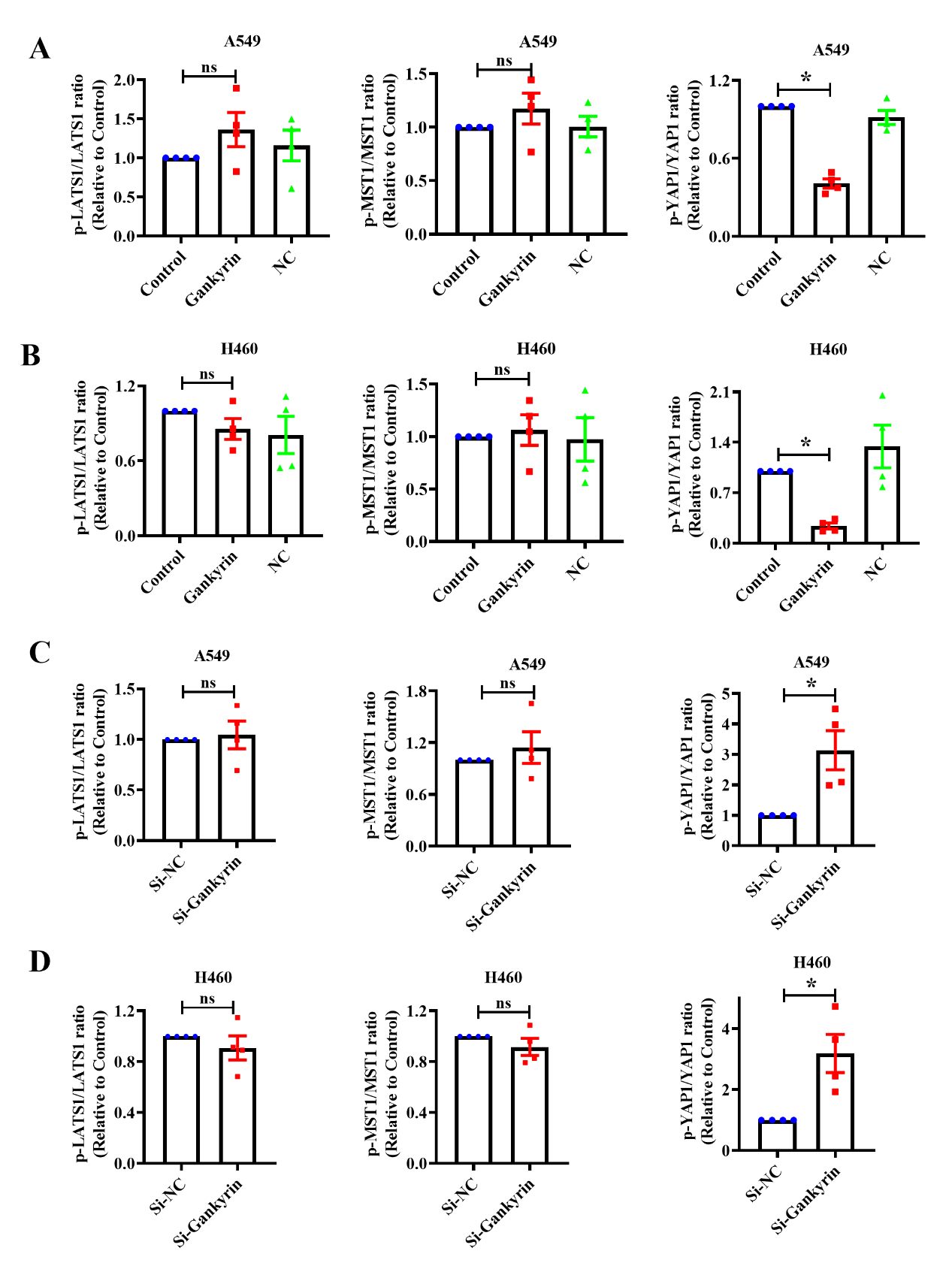


**Figure S1. Gankyrin inhibits the phosphorylation of YAP1, but not affects the phosphorylation of MST1 and LATS1.**

**(A-B)** The ratio of p-LATS/LATS1, p-MST1/MST1 and p-YAP1/YAP1 in A549 and H460 cells with overexpressing Gankyrin (n = 4, **P* < 0.05). **(C-D)** The ratio of p-LATS/LATS1, p-MST1/MST1 and p-YAP1/YAP1 in A549 and H460 cells with silencing Gankyrin (n = 4, **P* < 0.05).

**Supplementary figure S2**


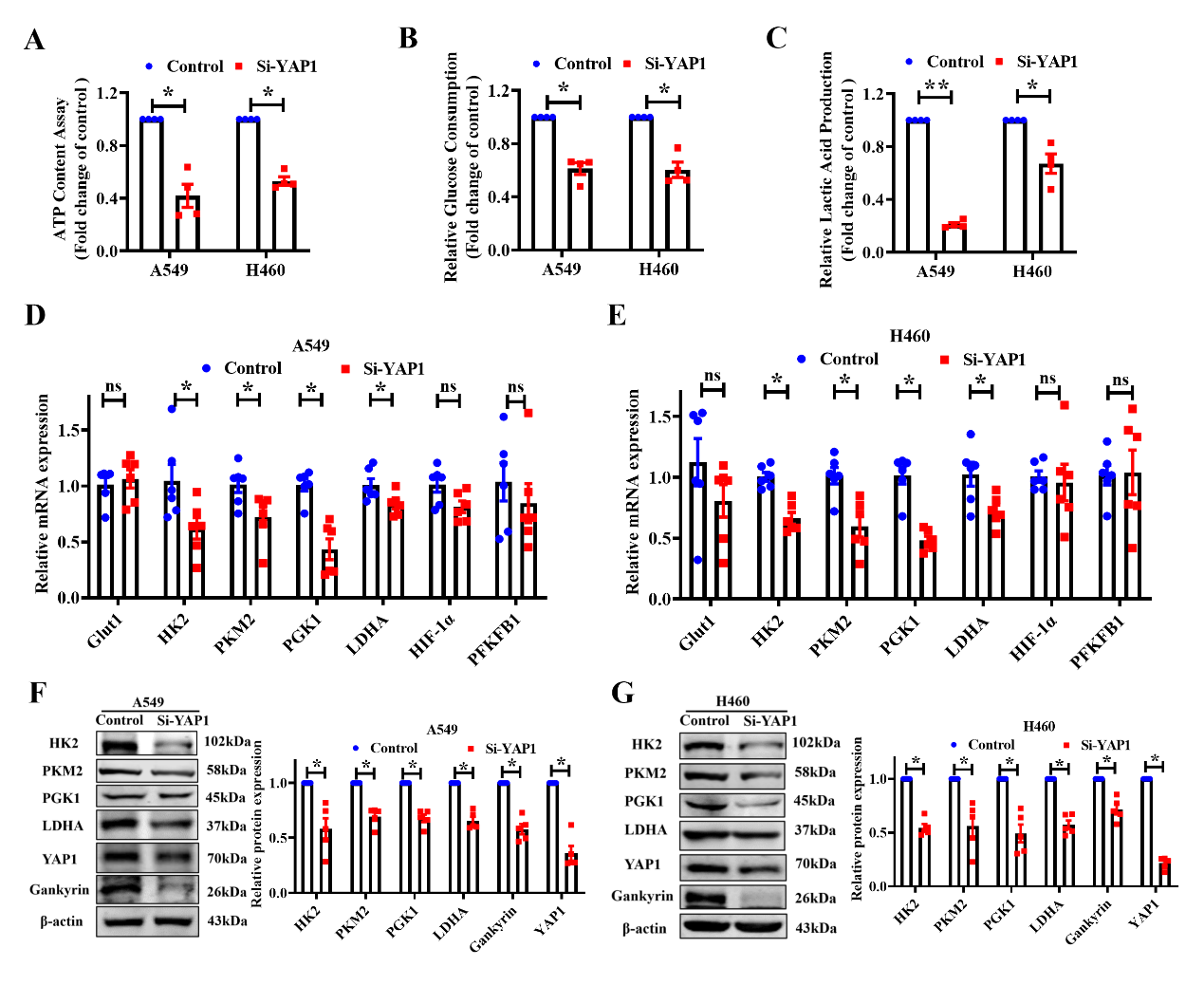


**Figure S2. Silencing YAP1 inhibits glycolysis in A549 and H460 cells.**

**(A)** ATP content assay was performed in both A549 and H460 cells (n = 4, **P* < 0.05). **(B)** Relative glucose consumption was determined in A549 and H460 cells (n = 4, **P* < 0.05). **(C)** Relative lactate production was measured in A549 and H460 cells, respectively (n = 4, **P* < 0.05, ***P* < 0.01). **(D-E)** qRT-PCR assay results show the mRNA levels of glycolytic genes including *Glut1*, *HK2*, *PKM2*, *PGK1*, *LDHA*, *HIF-1α,* and *PFKFB1* in A549 and H460 cells (n = 6, **P* < 0.05). **(F-G)** Western blotting results show the protein expression of HK2, PKM2, PGK1, and LDHA in A549 and H460 cells (n = 5, **P* < 0.05).

**Supplementary table S1**

**Table S1 Primer sequences used in qRT-PCR**

| Gene | Primer | Sequence (5’-3’) |
| --- | --- | --- |
| Human YAP1 | F | TGACCCTCGTTTTGCCATGA |
|  | R | GTTGCTGCTGGTTGGAGTTG |
| Human Gankyrin | F | GCAGGTTGGTCTCCTCTTCA |
|  | R | AATGGTCCTTAGCATCTGGA |
| Human E-cadherin | F | GAAACTCTCTCGGTCCA |
|  | R | GGAAACTCTCTCGGTCCA |
| Human N-cadherin | F | CGGGTAATCCTCCCAAATCA |
|  | R | CTTTATCCCGGCGTTTCATC |
| Human Vimentin | F | AACTTAGGGGCGCTCTTGTC |
|  | R | GGTGGACGTAGTCACGTAGC |
| Human ACTB | F | GGGAAATCGTGCGTGACATT |
|  | R | GGAACCGCTCATTGCCAAT |
| Human GLUT1 | F | CTTGGCTCCCTGCAGTTTG |
|  | R | GGACCCATGTCTGGTTGTAG |
| Human HK2 | F | TTGACATGGGCTCACTGAAC |
|  | R | CATCTTCACCAGGATAAGCCTC |
| Human PKM2 | F | GTGCCGCCTGGACATTGATTCA |
|  | R | AGTTCAGACGAGCCACATTCATTCC |
| Human PGK1 | F | GAGCCAAGTCGGTAGTCCTTATGAG |
|  | R | CACAGTCCTTCAAGAACAGAACATCCT |
| Human LDHA | F | CTTCTAAAGGAAGAACAGACCC |
|  | R | CAAGAGCAAGTTCATCTGCC |
| Human Hif-1α | F | AGCACAGTTACAGTATTCCAGCAGAC |
|  | R | TCATCAGTGGTGGCAGTGGTAGT |
| Human PFKFB1 | F | TGGACCTGGAGAAGCTGC |
|  | R | ACATAAATGCCCATGCGCG |
