# Supplementary figures and images for: Gankyrin modulated non-small cell lung cancer progression via glycolysis metabolism in a YAP1-dependent manner
Source: Cell Death Discov. 2022 Jul 9;8:312. doi: 10.1038/s41420-022-01104-3 (PMC9271063; doi:10.1038/s41420-022-01104-3)

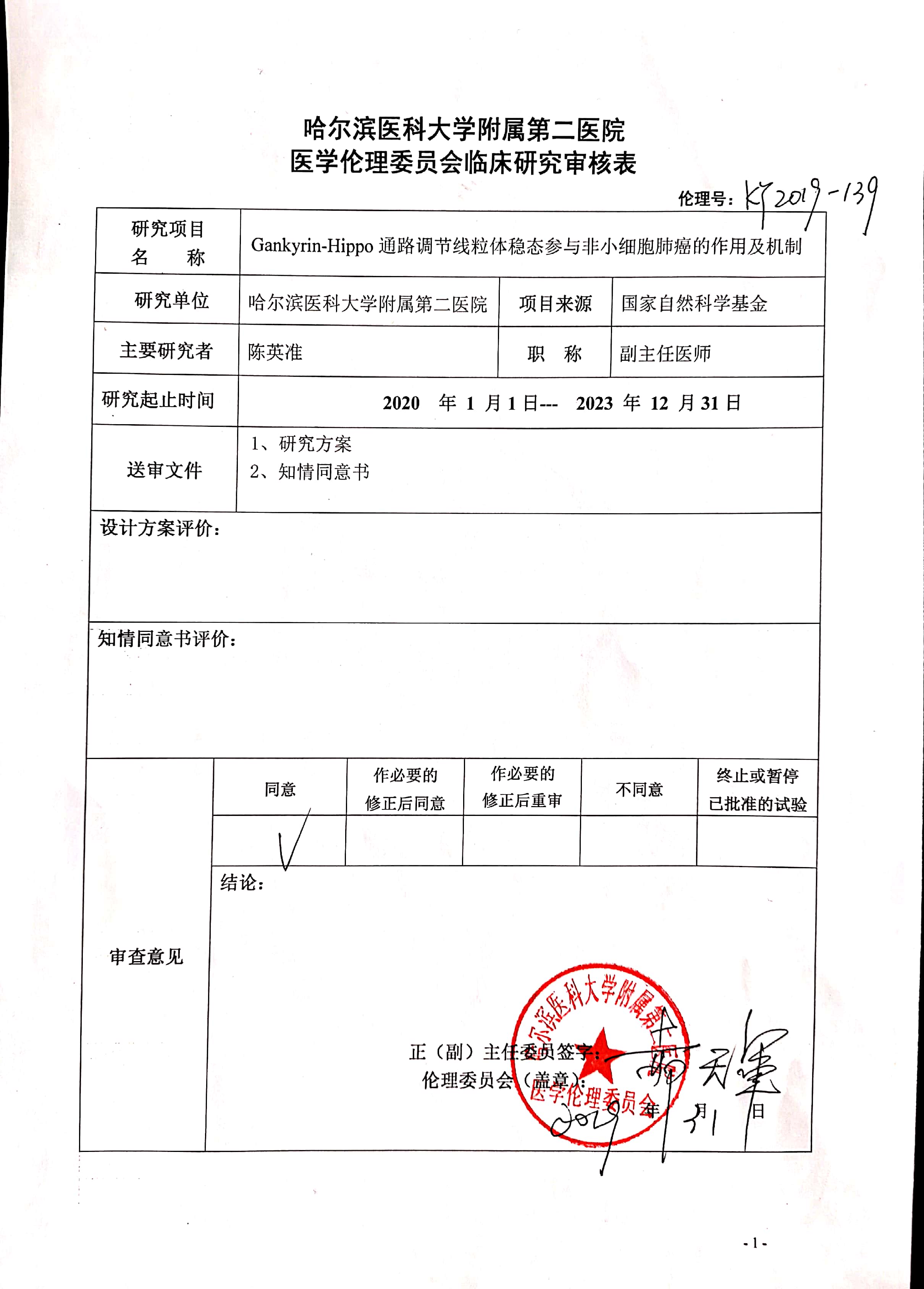

Supplement: Supplementary file 3 — Clinical sample Ethics statement [file 41420_2022_1104_MOESM3_ESM.jpg]

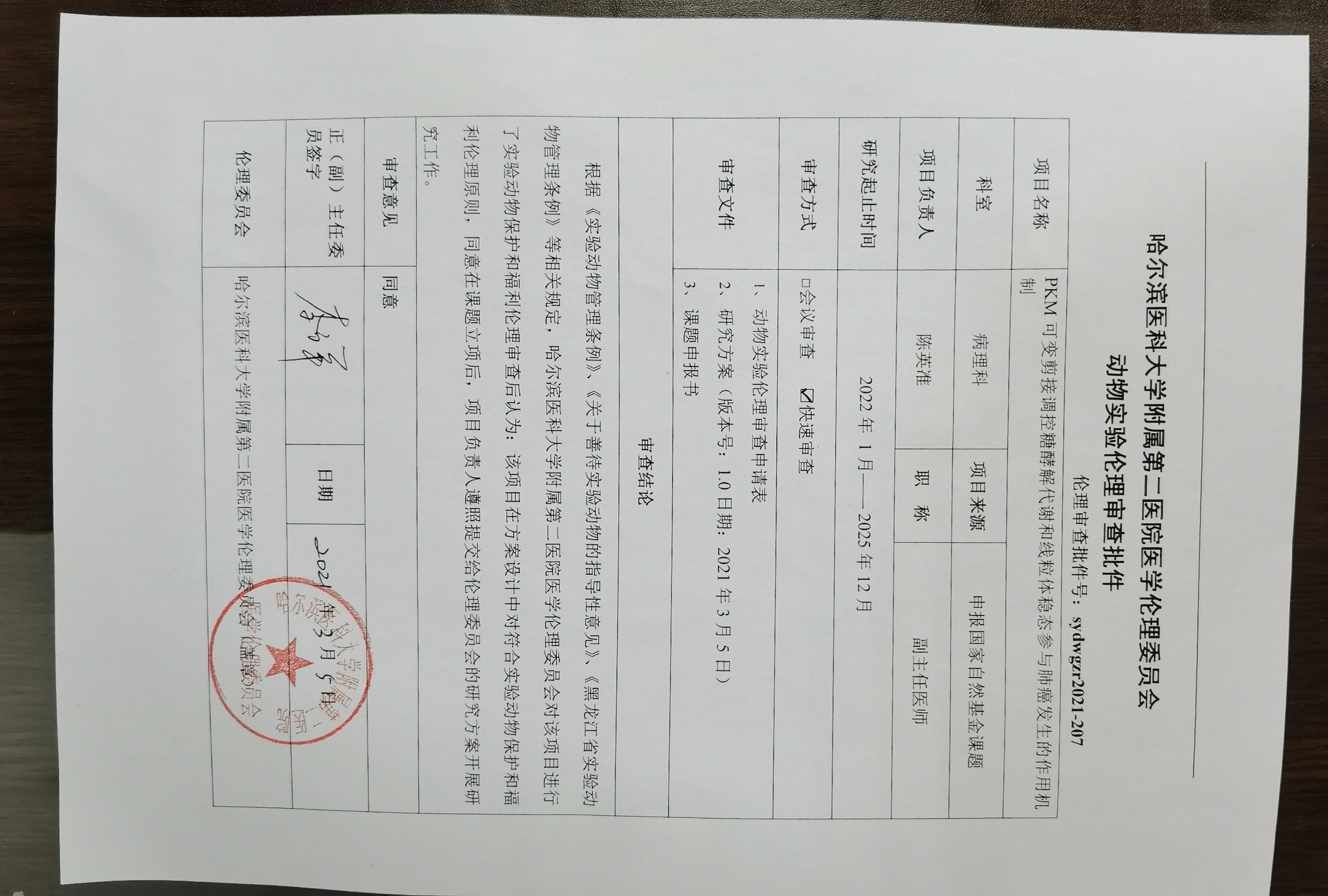

Supplement: Supplementary file 4 — Animal Ethics statement [file 41420_2022_1104_MOESM4_ESM.jpg]
