# Supplementary material for: Gankyrin modulated non-small cell lung cancer progression via glycolysis metabolism in a YAP1-dependent manner
Source: Cell Death Discov. 2022 Jul 9;8:312. doi: 10.1038/s41420-022-01104-3 (PMC9271063; doi:10.1038/s41420-022-01104-3)
Supplement: Supplementary file 5 — Editing Certificate [file 41420_2022_1104_MOESM5_ESM.pdf]

CERTIFICATE OF ENGLISH EDITING

This document certifies that the paper listed below has been edited to ensure that the language is clear and free of errors. The logical presentation of ideas and the structure of the paper were also checked during the editing process. The edit was performed by professional editors at Editage, a division of Cactus Communications, in cooperation with Taylor & Francis Group. The intent of the author's message was not altered in any way during the editing process. The quality of the edit has been guaranteed, with the assumption that our suggested changes have been accepted and have not been further altered without the knowledge of our editors.

Title

Gankyrin modulated non-small cell lung cancer progression via glycolysis metabolism in a YAP1-dependent manner.

Authors

Tong Yu, Yanyan Liu, Junwen Xue, Xiang Sun, Di Zhu, Lu Ma, Yingying Guo, Tongzhu Jin, Huiying Cao, Yingzhun Chen, Tong Zhu, Xuelian Li, Haihai Liang, Du Zhimin, Hongli Shan

Order No.

OOQFA_1

EDITINGSERVICES
Supporting Taylor & Francis authors

Signature

Vikas Narang

Vikas Narang,
Chief Operating Officer,
Editage

INFORMATION SECURITY MANAGEMENT SYSTEM DNV·GL ISO/IEC 27001

editage

Date of Issue
April 02, 2022
